# Neutrophil‐to‐lymphocyte ratio: link to congestion, inflammation, and mortality in outpatients with heart failure

**DOI:** 10.1002/ehf2.15240

**Published:** 2025-03-02

**Authors:** Asma O.M. Rezig, Moustafa I. Morsy, Elisabetta Caiazzo, Antonio Iaconelli, Armando Ialenti, David Hunter, Joe J. Cuthbert, Syed Kazmi, Tomasz J. Guzik, Dario Bruzzese, John G.F. Cleland, Andrew L. Clark, Pasquale Maffia, Pierpaolo Pellicori

**Affiliations:** ^1^ School of Infection and Immunity, College of Medical, Veterinary and Life Sciences University of Glasgow Glasgow UK; ^2^ Department of Pharmacy, School of Medicine and Surgery University of Naples Federico II Naples Italy; ^3^ School of Cardiovascular and Metabolic Health, College of Medical, Veterinary and Life Sciences University of Glasgow Glasgow UK; ^4^ Fondazione Policlinico Universitario Agostino Gemelli IRCCS Rome Italy; ^5^ Department of Cardiorespiratory Medicine, Centre for Clinical Sciences, Hull York Medical School University of Hull Kingston Upon Hull UK; ^6^ Department of Cardiology, Castle Hill Hospital Hull University Teaching Hospitals Trust Kingston Upon Hull UK; ^7^ Centre for Cardiovascular Science, Queen's Medical Research Institute University of Edinburgh Edinburgh UK; ^8^ Department of Internal and Agricultural Medicine and Omicron Medical Genomics Laboratory Jagiellonian University Medical College Kraków Poland; ^9^ Department of Public Health, School of Medicine and Surgery University of Naples Federico II Naples Italy; ^10^ Africa‐Europe CoRE in Non‐Communicable Diseases and Multimorbidity African Research Universities Alliance (ARUA), The Guild of European Research‐intensive Universities Glasgow UK

**Keywords:** chronic heart failure, congestion, inflammation, mortality, NLR

## Abstract

**Background:**

The neutrophil‐to‐lymphocyte ratio (NLR) may be a useful marker of inflammation, but its associations with clinical characteristics, signs of congestion and outcome in patients with chronic heart failure (HF) are unknown.

**Methods and results:**

We enrolled 4702 ambulatory patients with HF and either left ventricular systolic dysfunction or high N‐terminal pro‐B‐type natriuretic peptide (NTproBNP) (≥125 ng/L). Compared with those in the lowest quartile of NLR (≤2.05), patients in the highest quartile (≥4.10) were older, had higher NTproBNP, and were more likely to have HF with reduced left ventricular ejection fraction (HFrEF), atrial fibrillation and to be treated with loop diuretics.

In 813 patients with detailed echocardiographic assessment, lymphocyte count correlated inversely with NTproBNP (*r* = −0.31) and markers of congestion [left atrial volume index (*r* = −0.25), inferior vena cava diameter (*r* = −0.24)]; neutrophil count correlated positively with high‐sensitivity C‐reactive protein (hsCRP) (*r* = 0.31, *P* < 0.001).

During a median follow‐up of 54 (29–100) months, 3015 (64%) patients died. In models adjusted for NTproBNP and HsCRP, higher NLR [hazard ratio (HR):1.05; 95% confidence interval (CI) 1.03–1.06] and neutrophil count (HR:1.07; 95%CI 1.04–1.10) were associated with higher mortality rates; higher lymphocyte count (HR:0.88; 95%CI 0.82–0.95) was associated with lower risk (all *P* < 0.001).

**Conclusions:**

Low lymphocyte count is associated with more congestion and high neutrophil count with more inflammation, which may explain why a greater NLR is associated with a poorer prognosis. For patients with heart failure, NLR or its components could be useful for risk stratification or for monitoring evolving risk, but might also be therapeutic targets.

## Graphical abstract

Association of Neutrophil Count, Lymphocyte Count and Neutrophil‐to‐Lymphocyte ratio (NLR) with All‐cause Mortality. We enrolled 4702 ambulatory patients referred to a local HF clinic in the UK (The Hull LifeLab), of whom 813 had detailed echocardiographic assessment (top left). Lymphocyte count correlated inversely with NTproBNP (*r* = −0.31) and markers of congestion (left atrial volume index (*r* = −0.25), inferior vena cava diameter (*r* = −0.24) and positively with right ventricular function (TAPSE); neutrophil count correlated positively with hsCRP (*r* = 0.31, *P*<0.001) (bottom left). On the right panel, we report the adjusted hazard ratios (HR) per 1 unit change assessed over the complete follow‐up duration with corresponding forest plots for visualization. Further right, we assessed model discrimination at 2 years of follow‐up using the C‐index. ΔC‐index represents the difference between the “C‐index of the fully adjusted model + neutrophil count/lymphocyte count/NLR” and the “C‐index of the fully adjusted model alone”. Abbreviations used; NLR: neutrophil‐to‐lymphocyte ratio, BMI: body mass index, HR: heart rate, DM: diabetes mellitus, IHD: ischaemic heart disease, NYHA: New York heart association classification, NTproBNP: N‐Terminal Pro‐B type natriuretic peptide, hsCRP: high sensitivity C‐reactive protein, C‐index: concordance index, LVEF: left ventricular ejection fraction, HFrEF: heart failure with reduced left ventricular ejection fraction, HFmrEF: heart failure with mildly reduced left ventricular ejection fraction. HFpEF: heart failure with preserved left ventricular ejection fraction.

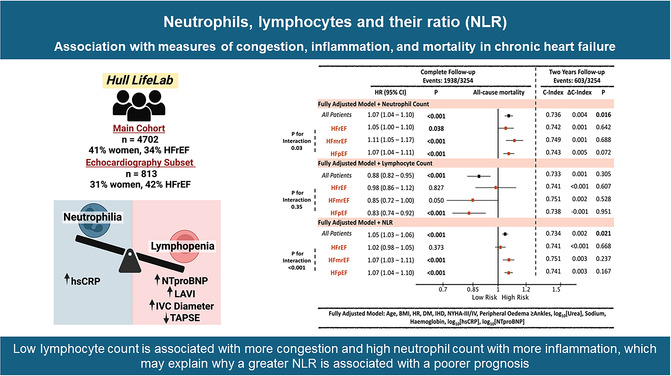

## Introduction

Inflammation and immune activation are involved in the pathophysiology of heart failure (HF) and are associated with many conditions that contribute to its development, including hypertension, diabetes, chronic kidney disease, obesity and ischaemic heart disease (IHD).^1^ Elevated circulating levels of cytokines and inflammatory markers, such as high‐sensitivity C‐reactive protein (hsCRP), are common in patients with HF and are associated with worse outcomes.^2^ Treatments that reduce inflammation might lower the risk of cardiovascular events in patients with IHD and elevated CRP,^3^, ^4^ but have limited benefit, or even cause harm, when HF has already developed.^1^, ^5^ This suggests that inflammation and immune activation may play different roles as HF develops.

White blood cell (WBC) counts are routinely measured and widely available in clinical practice. Observational studies suggest that a high WBC count is associated with a higher incidence of HF in patients with IHD and diabetes^6^ and in the general population.^7^ The ratio between neutrophil and lymphocyte count [neutrophil‐to‐lymphocyte ratio (NLR)] also predicts risk for patients admitted to hospital with HF.^8^ However, the association of neutrophils, lymphocytes and their ratio with indices of inflammation, disease severity and congestion and mortality in a large, well‐characterised population of ambulatory patients with HF has not been reported.

## Methods

### Study population

The Hull LifeLab collects demographic, clinical, biochemical and echocardiographic data from patients referred to a community HF clinic for assessment and management of suspected HF or with a pre‐existing diagnosis. The clinic serves a population of approximately 600 000.

In the present analysis, we have only included patients with symptoms or signs of chronic HF and either impaired left ventricular systolic function on echocardiography or a raised N‐terminal pro‐B‐type natriuretic peptide (NTproBNP), ≥125 ng/L according to current European Society of Cardiology (ESC)‐HF guidelines.^9^ Impaired left ventricular systolic function was defined as left ventricular ejection fraction (LVEF) < 50% or at least mild‐to‐moderate left ventricular systolic impairment on visual assessment. Patients were categorised as those with reduced (HFrEF), mildly reduced (HFmrEF) and preserved left ventricular ejection fraction (HFpEF).^9^ Clinical signs of congestion were evaluated based on lung auscultation, assessment of jugular venous pressure, peripheral oedema and liver examination, as previously described.^10^ Patients (*n* = 34) with a potential haematological malignancy were excluded from the analysis.^11^ Patients were enrolled between 2000 and 2021 and, for those still alive, the censorship date was 13 November 2021. A subset of patients (*n* = 813) had a detailed echocardiographic assessment, including assessment of left ventricular systolic function by global longitudinal strain (GLS), left atrial volume and inferior vena cava (IVC) diameter. A single experienced operator (P. P.) blinded to clinical details retrospectively reviewed these echocardiograms, as previously described.^12^, ^13^

The study conformed to the principles outlined in the Declaration of Helsinki and was approved by relevant ethical bodies. All participants gave written informed consent for their data to be used for research.

### Outcomes

The primary outcome was all‐cause mortality. Cause and mode of death were adjudicated at regular intervals following an in‐house guideline, based on information available from the clinical and electronic records.^2^ When clinical information was not available, mode and cause of death were adjudicated as ‘unknown’.

### Statistical analysis

Continuous data are expressed as medians (25th and 75th percentiles) and categorical data as numbers and percentages. Patients were grouped by quartiles of neutrophil count, lymphocyte count and by their ratio; upper and lower deciles are also reported. Student and Welch *t*‐tests, one‐way ANOVA and Kruskal–Wallis tests were used to compare differences between continuous variables and the *χ* ^2^ test was used to compare differences between categorical variables. Correlations of neutrophil count, lymphocyte count and NLR with other variables were assessed using bivariate Spearman's rank correlation analysis.

The associations between neutrophil count, lymphocyte count and NLR with all‐cause mortality were illustrated using Kaplan–Meier curves with log‐rank statistic. Cox proportional hazards regression analysis was used to model survival. The proportional hazard model assumption was assessed using Schoenfeld residuals. Nonlinearity was assessed using Martingale residuals; NTproBNP, hsCRP and urea were logarithmically transformed [log_10_] to comply with the linearity assumption. Multicollinearity was assessed using the variance inflation factor (VIF), where values above 10 indicated potential multicollinearity between independent variables. A baseline multivariable model was constructed selecting variables associated with mortality (those with *P* < 0.05) in univariable analysis, including log_10_[NTproBNP] and log_10_[hsCRP]. Neutrophil count, lymphocyte count and NLR were entered separately into the multivariable model. We did not impute missing values. Model performance for all‐cause mortality was assessed using the concordance index (C‐index) at 2 years of follow‐up; the significance of the increment change in C‐index due to the addition of neutrophil count, lymphocyte count and NLR to the multivariable model was assessed using the *compareC* function in R. All statistical analyses were performed using SPSS V.28 and R Statistical Software version (4.2.2). A two‐tailed *P* of <0.05 was considered significant in all analyses.

## Results

### Main cohort

#### Patient characteristics

Among the 4702 patients, the median age was 76 (68–82) years, 41% were women, and 34% had HFrEF. Compared with other phenotypes, patients with HFrEF were younger, more likely to be men and to be treated with a loop diuretic and less likely to have atrial fibrillation. They had higher NTproBNP, neutrophil count and NLR, but lymphocyte count and hsCRP were similar to the other HF phenotypes (*Table* S1).

Compared with patients in the lowest quartile of neutrophil count (≤3.75), those in the highest quartile (≥5.93) were more likely to have diabetes, HFrEF and AF and had higher NTproBNP (regardless of heart rhythm) and higher hsCRP (*Table* S2). Compared with patients in the lowest quartile of lymphocyte count (≤1.24), those in the highest quartile (≥2.14) were younger, more likely to be women, had greater body mass index (BMI) and were more likely to have diabetes but were less likely to have AF and had lower NTproBNP (regardless of heart rhythm) and hsCRP (*Table* S3).

Compared with the lowest quartile of NLR (≤2.05), patients in the highest quartile (≥4.10) were older, were more likely to be in AF, had higher NTproBNP (regardless of heart rhythm) and hsCRP and were more likely to have more severe symptoms and signs of congestion and to be treated with loop diuretics (*Table* 1).

**Table 1:** Baseline characteristics of patients with heart failure stratified by quartiles of neutrophil‐to‐lymphocyte ratio

Variable		Missing^a^	Decile 1 ≤ 1.53	Quartile 1 ≤ 2.05	Quartile 2 2.06–2.89	Quartile 3 2.90–4.09	Quartile 4 ≥ 4.10	Decile 10 ≥ 6.03	*P*
			*N* = 470	*N* = 1174	*N* = 1174	*N* = 1175	*N* = 1174	*N* = 470	
Demographics									
Age (years)		0 (0)	72 (64–78)	73 (65–80)	76 (68–82)	76 (70–82)	78 (71–83)	78 (72–84)	<0.001
Sex (women)		0 (0)	202 (43)	529 (45)	477 (41)	458 (39)	446 (38)	182 (39)	0.003
Diabetes, *n*. (%)		0 (0)	98 (21)	250 (21)	268 (23)	301 (26)	315 (27)	111 (24)	0.006
Hypertension, *n*. (%)		0 (0)	209 (44)	558 (48)	574 (49)	537 (46)	526 (45)	190 (40)	0.19
IHD, *n*. (%)		0 (0)	190 (40)	463 (39)	493 (42)	461 (39)	480 (41)	194 (41)	0.48
COPD, *n*. (%)		0 (0)	28 (6)	79 (7)	105 (9)	117 (10)	153 (13)	70 (15)	<0.001
BMI (kg/m^2^)		18 (<1)	29.0 (25.6–33.2)	28.8 (25.3–32.9)	28.5 (24.8–32.4)	27.9 (24.4–32.1)	27.0 (23.8–31.2)	26.2 (22.6–30.2)	<0.001
Systolic BP (mmHg)		6 (<1)	141 (124–159)	140 (123–159)	141 (124–160)	139 (121–158)	137 (118–156)	133 (115–151)	<0.001
Diastolic BP (mmHg)		5 (<1)	80 (71–89)	80 (70–89)	78 (70–88)	77 (68–88)	76 (66–85)	73 (64–84)	<0.001
Clinical examination—Symptoms and signs									
Peripheral oedema ≥ ankles, *n*. (%)		229 (5)	19 (4)	55 (5)	79 (7)	116 (10)	208 (19)	99 (22)	<0.001
Lung crackles, *n*. (%)		419 (9)	30 (7)	97 (9)	125 (12)	151 (14)	242 (22)	119 (28)	<0.001
Raised JVP, *n*. (%)		474 (10)	38 (9)	114 (11)	137 (13)	171 (16)	279 (26)	136 (32)	<0.001
Liver distension, *n*. (%)		1750 (37)	8 (3)	14 (2)	20 (3)	29 (4)	62 (8)	37 (12)	<0.001
NYHA III/IV, *n*. (%)		0 (0)	74 (16)	220 (19)	269 (23)	371 (32)	477 (41)	219 (47)	<0.001
ECG									
Heart rate (bpm)		4 (<1)	70 (60–81)	71 (61–82)	72 (61–84)	75 (64–88)	78 (66–91)	79 (68–94)	<0.001
Atrial fibrillation, *n*. (%)		62 (1)	118 (25)	329 (28)	395 (34)	421 (36)	519 (45)	215 (47)	<0.001
QRS width (ms)		169 (4)	98 (86–114)	98 (86–116)	100 (88–122)	100 (88–126)	100 (88–124)	102 (86–126)	0.002
Echocardiography									
HF phenotype	HFrEF	0 (0)	154 (33)	367 (31)	367 (31)	444 (38)	432 (37)	190 (40)	0.001
	HFmrEF		91 (19)	238 (20)	261 (22)	224 (19)	246 (21)	93 (20)	
	HFpEF		225 (48)	569 (48)	546 (46)	507 (43)	496 (42)	187 (40)	
LVEDD (cm)		743 (16)	5.2 (4.6–6.0)	5.2 (4.6–5.9)	5.2 (4.7–5.9)	5.3 (4.7–6.0)	5.2 (4.6–5.9)	5.2 (4.6–6.0)	0.432
LAD (cm)		700 (15)	4.0 (3.6–4.5)	4.0 (3.6–4.5)	4.1 (3.7–4.6)	4.2 (3.7–4.7)	4.2 (3.8–4.8)	4.3 (3.8–4.9)	<0.001
Mitral regurgitation ≥ mild		648 (14)	263 (64)	650 (63)	697 (68)	731 (73)	756 (76)	304 (77)	<0.001
Blood tests									
NTproBNP (ng/L)	Overall	513 (11)	610 (257–1391)	751 (290–1612)	1019 (425–2245)	1310 (568–3160)	1829 (789–4014)	2366 (975–5330)	<0.001
	SR		364 (207–989)	462 (229–1066)	674 (310–1708)	878 (314–2348)	1176 (483–3211)	1562 (589–4498)	<0.001
	AF		1483 (940–2146)	1466 (928–2365)	1768 (986–2866)	2101 (1181–4093)	2522 (1456–4579)	3100 (1614–6450)	<0.001
Serum creatinine (μmol/L)		223 (5)	89 (76–107)	90 (76–108)	98 (80–120)	100 (82–124)	106 (82–136)	107 (84–143)	<0.001
eGFR (mL/min/1.73 m^2^)		223 (5)	65 (52–79)	64 (52–77)	59 (44–72)	57 (44–72)	54 (39–69)	51 (38–67)	<0.001
Urea (mmol/L)		124 (3)	6.3 (4.9–8.2)	6.4 (5.1–8.1)	7.0 (5.4–9.6)	7.2 (5.5–10.0)	8.1 (6.1–11.7)	8.4 (6.5–12.5)	<0.001
Albumin (g/L)		344 (7)	38 (36–40)	38 (36–40)	38 (36–40)	37 (35–40)	36 (33–39)	35 (32–38)	<0.001
Haemoglobin (g/dL)	All	123 (3)	13.6 (12.5–14.7)	13.7 (12.5–14.7)	13.5 (12.3–14.5)	13.2 (12.0–14.4)	12.6 (11.4–14.0)	12.5 (11.2–13.9)	<0.001
	Women		13.0 (12.1–13.8)	13.0 (12.1–13.9)	12.9 (11.8–13.7)	12.6 (11.6–13.6)	12.4 (11.3–13.4)	12.3 (11.3–13.5)	<0.001
	Men		14.2 (13.1–15.0)	14.2 (13.1–15.1)	14.0 (12.8–14.9)	13.7 (12.3–14.8)	12.8 (11.4–14.2)	12.7 (11.2–14.1)	<0.001
WBC count (×10^9^/L)		0 (0)	6.6 (5.7–7.9)	6.8 (5.8–8.0)	7.2 (6.0–8.4)	7.5 (6.3–8.8)	8.4 (6.9–10.3)	9.1 (7.5–11.6)	<0.001
Neutrophil count (×10^9^/L)		5 (<1)	3.24 (2.69–3.88)	3.60 (2.97–4.35)	4.45 (3.73–5.24)	5.02 (4.19–5.99)	6.32 (5.09–8.03)	7.36 (5.92–9.45)	‐
Lymphocyte count (×10^9^/L)		0 (0)	2.61 (2.16–3.06)	2.32 (1.89–2.79)	1.82 (1.51–2.18)	1.48 (1.23–1.78)	1.08 (0.84–1.36)	0.86 (0.66–1.12)	‐
Monocyte count (×10^9^/L)		1 (<1)	0.60 (0.47–0.72)	0.60 (0.48–0.74)	0.63 (0.51–0.79)	0.66 (0.53–0.82)	0.68 (0.53–0.87)	0.70 (0.52–0.89)	<0.001
Eosinophil count (×10^9^/L)		86 (2)	0.17 (0.11–0.26)	0.18 (0.11–0.26)	0.17 (0.11–0.27)	0.16 (0.10–0.25)	0.13 (0.07–0.21)	0.09 (0.04–0.17)	<0.001
Basophil count (×10^9^/L)		176 (4)	0.030 (0.020–0.040)	0.030 (0.020–0.040)	0.030 (0.020–0.040)	0.030 (0.020–0.040)	0.030 (0.020–0.040)	0.030 (0.020–0.040)	0.007
hsCRP (mg/L)		908 (19)	2.7 (1.2–5.6)	2.7 (1.3–5.6)	3.7 (1.6–7.0)	4.6 (1.7–9.8)	7.3 (2.9–19.0)	8.8 (3.4–24.0)	<0.001
Treatment at time of referral									
Loop diuretic, *n*. (%)		0 (0)	248 (53)	616 (52)	710 (60)	771 (66)	830 (71)	335 (71)	<0.001
>40‐mg furosemide/day, *n*. (%)		0 (0)	67 (14)	179 (15)	272 (23)	316 (27)	396 (34)	165 (35)	<0.001
Beta‐blocker, *n*. (%)		0 (0)	300 (64)	728 (62)	730 (62)	675 (57)	617 (53)	233 (50)	<0.001
ACEi, *n*. (%)		0 (0)	268 (57)	656 (56)	640 (54)	654 (56)	662 (56)	254 (54)	0.83
ARB, *n*. (%)		0 (0)	69 (15)	149 (13)	153 (13)	144 (12)	127 (11)	45 (10)	0.37
MRA, *n*. (%)		0 (0)	83 (18)	192 (16)	208 (18)	233 (20)	232 (20)	90 (19)	0.083

Abbreviations: ACEi, angiotensin‐converting enzyme inhibitor; ARB, angiotensin receptor blocker; BMI, body mass index; BP, blood pressure; COPD, chronic obstructive pulmonary disease; eGFR, estimated glomerular filtration rate; HF, heart failure; HFmrEF, heart failure with mildly reduced ejection fraction; HFpEF, heart failure with preserved ejection fraction; HFrEF, heart failure with reduced ejection fraction; hsCRP, high‐sensitivity C‐reactive protein; IHD, ischaemic heart disease; JVP, jugular vein pressure; LAD, left atrial diameter; LVEDD, left ventricular end‐diastolic diameter; MRA, mineralocorticoid receptor antagonist; NTproBNP, N‐terminal pro‐B‐type natriuretic peptide; NYHA, New York Heart Association; WBC, white blood cell.

^a^ Missing refers to missing values from the overall included patients, *n* = 4702.

#### Correlations with congestion and inflammation

**Figure 1:**
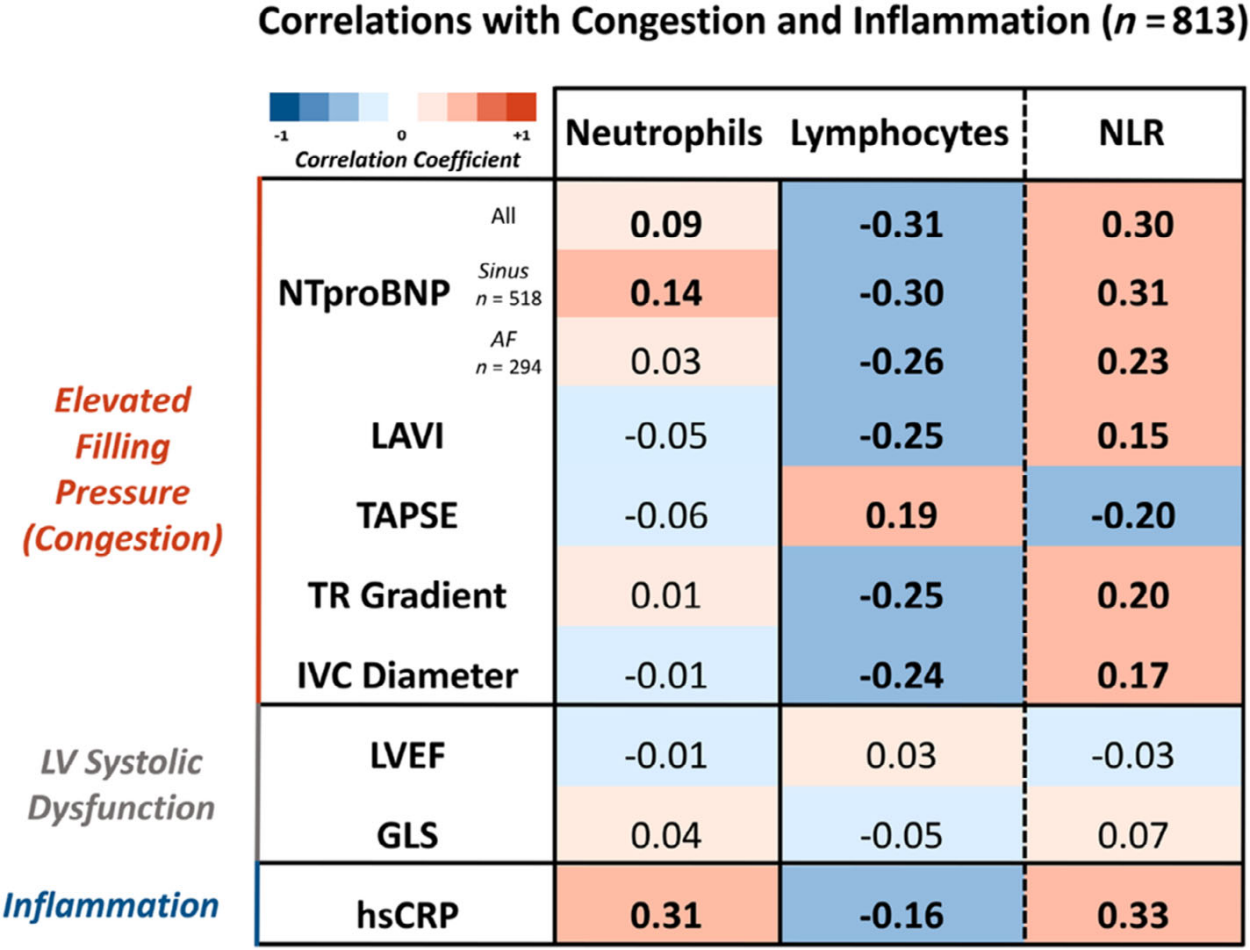
Associations with congestion, inflammation and cardiac function. In a subset of 813 patients with a detailed echocardiographic examination, lymphocyte count, but not neutrophil count, inversely correlated with markers of elevated filling pressures, such as left atrial volume and inferior vena cava diameter; NLR did not correlate with measures of left ventricular systolic function. Significant (*P* < 0.05) associations using bivariate Spearman's rank correlation analysis are highlighted in bold. GLS, global longitudinal strain; hsCRP, high‐sensitivity C‐reactive protein; IVC diameter, inferior vena cava diameter; LAVI, left atrial volume indexed for body surface area; TAPSE, tricuspid annular plane systolic excursion; LVEF, left ventricular ejection fraction; NLR, neutrophil‐to‐lymphocyte ratio; NTproBNP, N‐Terminal Pro‐B type natriuretic peptide; TR gradient, trans‐tricuspid systolic gradient; AF, atrial fibrillation.

In a subset of 813 patients who had detailed echocardiographic images [median age 75 (66–81) years, 31% women, 42% with LVEF <40%; *Table*s S4 and S5], neutrophil count correlated positively with hsCRP (*r* = 0.31, *P* < 0.001). Lymphocyte count correlated negatively with NTproBNP (*r* = −0.31, *P* < 0.001), left atrial volume index (LAVI) (*r* = −0.25, *P* < 0.001), trans‐tricuspid systolic gradient (TR gradient) (*r* = −0.25, *P* < 0.001) and IVC diameter (*r* = −0.24, *P* < 0.001) but positively with tricuspid annular plane systolic excursion (TAPSE) (*r* = 0.19, *P* < 0.001). NLR correlated positively with hsCRP (*r* = 0.33, *P* < 0.001), NTproBNP (*r* = 0.30, *P* < 0.001), LAVI (*r* = 0.15, *P* < 0.001), TR gradient (*r* = 0.20, *P* < 0.001) and IVC diameter (*r* = 0.17, *P* < 0.001) but negatively with TAPSE (*r* = −0.20, *P* < 0.001). There was no association between neutrophil count, lymphocyte count and their ratio with measures of left ventricular systolic function, including GLS and LVEF (*Figure* 1).

#### Outcome

**Figure 2:**
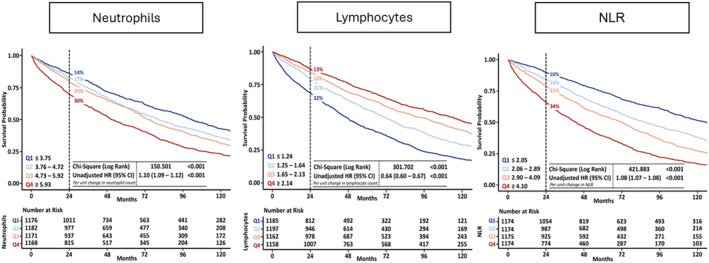
Associations of neutrophil count, lymphocyte count and neutrophil‐to‐lymphocyte ratio (NLR) with survival. Kaplan–Meier curves illustrating time to all‐cause mortality by quartiles of neutrophil count (left panel), lymphocyte count (middle panel) and NLR (right panel). At 2 years of follow‐up, patients in the highest quartile of NLR (≥4.10) and neutrophil count (≥5.93) had a mortality rate of 34% and 30% compared with 10% and 14% in the lowest quartiles, respectively. Patients in the lowest quartile of lymphocyte count (≤1.24) had a 32% mortality rate compared with 13% for patients in the highest quartile (≥2.14), *P* for all <0.001. Unadjusted hazard ratios (HR) and 95% confidence intervals (95% CI) for all‐cause mortality are presented per unit change.

Within 2 years, 951 (20%) patients died, of whom 524 (55%) died from cardiovascular causes; over the entire follow‐up [median 54 (29–100) months], we recorded 3015 (64%) deaths. Patients in the highest quartile of neutrophil count (≥5.93) had a 2 year mortality rate of 30% compared with 14% in the lowest quartile. Patients in the highest quartile of lymphocyte count (≥2.14) had a 13% 2 year mortality rate compared with 32% for patients in the lowest quartile (≤1.24). Patients with an NLR (≥4.10) had a 2 year mortality of 34% compared with 10% in the lowest quartile, *P* < 0.001 for all (*Figure* 2). Associations with survival stratified by LVEF phenotypes are shown in *Figure*s S1–S3. The proportion of non‐cardiovascular deaths was higher for patients in the highest quartile of neutrophil count (≥5.93; 46%) compared with the lowest quartile (≤3.75; 41%). The proportion of cardiovascular deaths was higher for patients in the lowest quartile of lymphocyte count (≤1.24; 56%) compared with the highest quartile (≥2.14; 50%) (Table 2). Patients with HFrEF and HFmrEF predominantly died of cardiovascular causes (*n* = 241, 66%; and *n* = 106, 54%, respectively); among those with HFpEF, 52% of deaths were for non‐cardiovascular causes (*n* = 202). Cause of death by quartiles of neutrophil count, lymphocyte count and NLR stratified by LVEF phenotype are shown in *Table*s S6–S8.

**Table 2:** Rate, cause and mode of death at 2 years by quartiles of neutrophil count, lymphocyte count and NLR.

	Neutrophil count					Lymphocyte count					Neutrophil‐to‐lymphocyte ratio				
	All	Q1	Q2	Q3	Q4	All	Q1	Q2	Q3	Q4	All	Q1	Q2	Q3	Q4
	4685	1173	1179	1170	1163	4690	1181	1195	1158	1156	4685	1172	1172	1172	1169
		≤3.75	3.76–4.72	4.73–5.92	≥5.93		≤1.24	1.25–1.64	1.65–2.13	≥2.14		≤2.05	2.06–2.89	2.90–4.09	≥4.10
2 year mortality	951 (20)	163 (14)	204 (17)	235 (20)	349 (30)	953 (20)	372 (32)	250 (21)	181 (16)	150 (13)	951 (20)	119 (10)	187 (16)	248 (21)	397 (34)
Cardiovascular	524 (55)	94 (58)	124 (61)	122 (52)	184 (53)	524 (55)	209 (56)	146 (58)	94 (52)	75 (50)	524 (55)	63 (53)	115 (62)	135 (54)	211 (53)
Sudden	274 (29)	46 (28)	64 (32)	72 (31)	92 (26)	274 (29)	95 (26)	80 (32)	58 (32)	41 (27)	274 (29)	31 (26)	63 (34)	77 (31)	103 (26)
Terminal HF	169 (18)	29 (18)	37 (18)	38 (16)	65 (19)	169 (18)	79 (21)	41 (16)	25 (14)	24 (16)	169 (18)	18 (15)	35 (19)	38 (15)	78 (20)
Other/CV	81 (8)	19 (12)	23 (11)	12 (5)	27 (8)	81 (8)	35 (9)	25 (10)	11 (6)	10 (7)	81 (8)	14 (12)	17 (9)	20 (8)	30 (7)
Non‐CV	408 (43)	67 (41)	78 (38)	103 (44)	160 (46)	410 (43)	154 (42)	100 (40)	84 (46)	72 (48)	408 (43)	56 (47)	66 (35)	108 (44)	178 (45)
Infection	171 (18)	28 (17)	38 (19)	42 (18)	63 (18)	172 (18)	66 (18)	47 (19)	34 (19)	25 (17)	171 (18)	26 (22)	25 (13)	42 (17)	78 (20)
Cancer	117 (12)	18 (11)	19 (9)	31 (13)	49 (14)	117 (12)	41 (11)	26 (10)	32 (17)	18 (12)	117 (12)	14 (12)	24 (13)	30 (12)	49 (12)
Other/non‐CV	120 (13)	21 (13)	21 (10)	30 (13)	48 (14)	121 (13)	47 (13)	27 (11)	18 (10)	29 (19)	120 (13)	16 (13)	17 (9)	36 (15)	51 (13)
Unknown	19 (2)	2 (1)	2 (1)	10 (4)	5 (1)	19 (2)	9 (2)	4 (2)	3 (2)	3 (2)	19 (2)	0 (0)	6 (3)	5 (2)	8 (2)
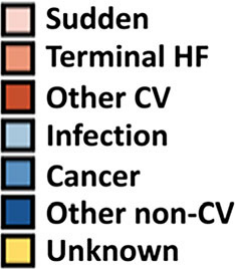	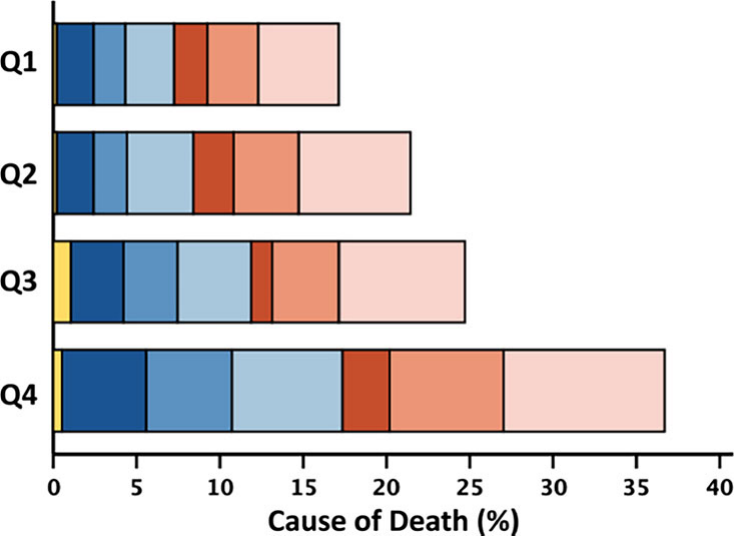					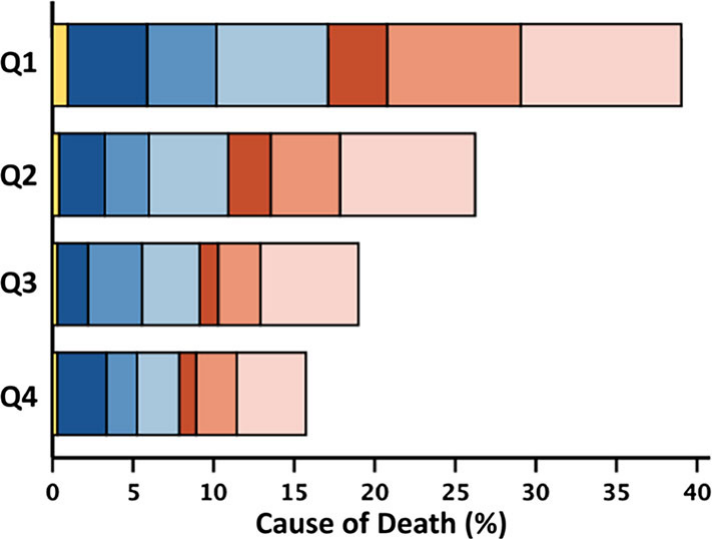					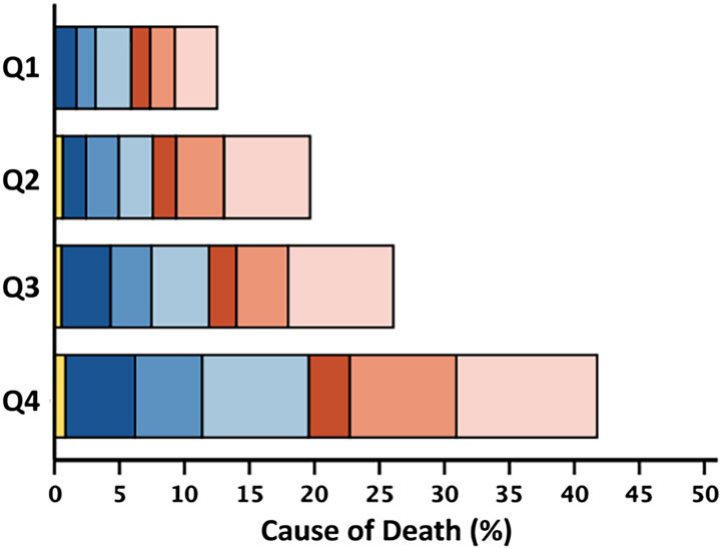				

*Note*: Two‐year all‐cause mortality expressed as a percentage of the total number of patients overall, and in respective quartiles. Cause‐of‐death rows represent the percentage of the total number of deaths in patients overall and in respective quartiles. The stacked bar figures show cause/mode of death by quartile of white cell variable, represented as a percentage of the total number of deaths in the overall population.

Abbreviations: HF, heart failure; CV, cardiovascular.

In univariate analysis, an increase of 1 × 10^9^ cells/L in neutrophil count was associated with a 10% greater mortality, and an increase in lymphocyte count of 1 × 10^9^ cells/L was associated with a 36% lower mortality. One unit increase in NLR was associated with an 8% higher mortality (*Figure* 2). In a fully adjusted model that included both log_10_[NTproBNP] and log_10_[hsCRP], adding either higher NLR [hazard ratio (HR): 1.05; 95% confidence interval (CI) 1.03–1.06] or neutrophil count (HR: 1.07; 95% CI 1.04–1.10) were associated with higher mortality while higher lymphocyte count (HR: 0.88; 95% CI 0.82–0.95) was associated with lower mortality (all *P* < 0.001). For patients with HFrEF compared with other phenotypes, neutrophil count, lymphocyte count and NLR were less strongly associated with mortality. In the overall population, the 2 year C‐index for all‐cause mortality was only marginally improved with the addition of either neutrophil count or NLR to the fully adjusted model (Graphical abstract).

## Discussion

We found that in ambulatory patients with HF, 2 year mortality rates are worse for patients with higher NLR or neutrophil count and lower lymphocyte count, even after adjusting for age, HF symptoms and signs, haemoglobin, hsCRP and NTproBNP. Neutrophil count was most strongly associated with hsCRP while lymphocyte count was inversely correlated with NTproBNP, left atrial volume index and IVC diameter. However, these associations were not strong.

In clinical practice, both lymphocyte and neutrophil counts are measured routinely as part of a full blood count and are thus widely available. NLR, the ratio of neutrophils to lymphocytes, is associated with poorer outcomes in many conditions, including HF.^14^, ^15^, ^16^ However, lymphocytes and neutrophils originate from different progenitor cells and have different roles in the immune response. Lymphocytes are effector cells of the adaptive (T lymphocytes and B lymphocytes) and innate (natural killer cells) immune system: they originate from the lymphoid progenitor cells in the bone marrow then differentiate into distinct phenotypes. Neutrophils are the most abundant white blood cell type in peripheral blood: they originate from the myeloid progenitor cells in the bone marrow, along with monocytes, red blood cells and megakaryocytes and are one of the first lines of defence against pathogens.^17^

In patients free of cardiovascular disease, high neutrophil count and low lymphocyte count are associated with a higher risk of cardiovascular events, including HF.^18^, ^19^ Interestingly, they have a different response to anti‐inflammatory treatments: in a recent pooled analysis of five trials in patients at high risk of, or with, established CVD, lipid‐lowering therapies such as rosuvastatin did not have a significant effect on the NLR, even if CRP fell; on the other hand, canakinumab, a monoclonal antibody that blocks and neutralises interleukin‐1 beta, reduced hsCRP and interleukin‐6 (IL‐6) levels and decreased NLR, mostly driven by a reduction on neutrophil count; but methotrexate increased NLR, mostly by decreasing lymphocytes.^20^ Ziltivekimab, a monoclonal antibody targeting interleukin‐6, reduced both hsCRP and NLR in patients with chronic kidney disease who had elevated hsCRP, but there was only a modest correlation between changes in NLR and hsCRP after 12 weeks of treatment (*r* = 0.26, *P* < 0.001).^21^ Clinical trials evaluating ziltivekimab in patients with HF are ongoing (NCT05636176). In healthy people, a short course of glucocorticoids has effects on lipid and glucose metabolism and might also reduce CRP but increase leukocytes, particularly neutrophils, and increase body weight and NT‐proBNP, perhaps reflecting water and salt retention.^22^, ^23^

Evidence on the mechanistic role of neutrophils and lymphocytes in HF development and progression is mainly derived from animal models.^1^ Although these models mimic the biological processes involved in the acute phase of myocardial damage, remodelling or haemodynamic stress, they do not address the multisystem nature of chronic HF in humans. In cohorts that enrolled patients with de novo or acute HF, NLR correlated with markers of both inflammation (IL‐6) and congestion (NTproBNP, bio‐adrenomedullin and CA‐125) and was associated with adverse prognosis and clinical status.^8^, ^24^ However, most studies of ambulatory patients with HF were small and almost exclusively enrolled patients with advanced HFrEF.^25^, ^26^ To the best of our knowledge, the present study is the largest of its kind to investigate the possible role of NLR in a large, well‐characterised cohort of ambulatory patients with HF, with a broad spectrum of LVEF, representative of clinical practice.

Markers of systemic inflammation, like hsCRP, and inflammatory cytokines, for instance, tumour necrosis factor‐alpha (TNF‐α) or IL‐6, are often elevated in patients with HF and associated with greater severity of cardiac dysfunction and worse prognosis.^2^, ^27^ In experimental conditions, the development of peripheral venous congestion activates the innate immune system and enhances the secretion of pro‐inflammatory cytokines even in healthy individuals.^28^ Inflammation is generally associated with micro‐vascular dysfunction, leading to increased permeability of capillaries and leakage of water, electrolytes and small molecules into the interstitial space; in other words, ‘congestion’. Right ventricular dysfunction might worsen intravascular and peripheral congestion, cause bowel oedema and alter gut permeability, allowing endotoxins to enter the circulation, causing an inflammatory response.^29^

Medicines known to improve outcomes in patients with HFrEF, such as renin–angiotensin–aldosterone system inhibitors or sodium‐glucose co‐transporter 2 inhibitors, have modest effects on reducing circulating markers of inflammation but can improve congestion.^30^, ^31^ In patients with worsening HF who developed signs of congestion, intensification of treatment with loop diuretics had a mixed effect on biomarkers of immune activation and inflammation, with normalisation of endotoxin, but not cytokine, levels.^32^ However, trials of agents specifically targeting inflammation in patients with HF were neutral or were stopped prematurely due to possible harm.^1^, ^5^

In summary, increasing NLR is associated with higher mortality, but neutrophils and lymphocytes are associated differentially with patient characteristics, measures of inflammation and congestion. A better understanding of the complex link between congestion, immune response and inflammation might allow for more targeted therapies and improved outcomes, particularly for patients with HFpEF.

### Limitations

This was a post‐hoc analysis of a longitudinal, prospective registry and therefore cannot address causality. Moreover, we only assessed NLR at baseline and did not study longitudinal changes in neutrophils or lymphocytes, inflammation or congestion that might have occurred with initiation or uptitration of guideline‐recommended treatments. Although we excluded patients with an absolute lymphocyte count ≥5 × 10^9^/L (~0.1%), which might suggest haematological malignancy, we do not have accurate records of neoplastic hematologic diseases or chronic inflammatory conditions and did not exclude patients who might have had an active infection when they attended their clinical visit. Additionally, even though we systematically collected cardiovascular therapies at each visit, we did not have detailed information on short‐term use in intervening periods or dose of additional (e.g., anti‐inflammatory/immunosuppressive) therapies, which might have modified neutrophils, lymphocytes or their ratio.

## Conclusions

Low lymphocyte count is associated with more evidence of congestion and increasing neutrophil count with more evidence of inflammation, which may explain why a greater NLR is associated with a poorer prognosis. For patients with heart failure, NLR or its components could be useful for risk stratification or for monitoring evolving risk, but might also be therapeutic targets.

## Conflict of interest statement

Dr. Pellicori has received consultancy honoraria and/or sponsorship support from Boehringer Ingelheim, Pharmacosmos, Novartis, Vifor, AstraZeneca and Caption Health and research support from Bristol Myers Squibb in the past 5 years, not connected with this manuscript. Dr. Cleland reports grants from British Heart Foundation, personal fees from Abbott, personal fees from Amgen, grants and personal fees from Bayer, grants and personal fees from Bristol Myers Squibb, personal fees from Novartis, personal fees from Medtronic, personal fees from Idorsia, grants and personal fees from Vifor, personal fees from Servier, personal fees and non‐financial support from Boehringer‐Ingelheim, personal fees from AstraZeneca, personal fees from Innolife, personal fees from Torrent, grants and personal fees from Johnson & Johnson, grants and personal fees from Myokardia, personal fees from Respicardia, grants and personal fees from Viscardia, personal fees and non‐financial support from NI Medical, grants from Pharma Nord, and grants from Pharmacosmos. Dr. Maffia has received consultancy honoraria from Pangea Botanica Ltd. and Orion Biotechnology not connected with this manuscript. Dr. Guzik reports consulting fees for Moderna. Others do not have any conflicts of interest to declare.

## Funding

J. G. F. C. is supported by the British Heart Foundation Centre of Research Excellence (RE/18/6134217). P. P. has received a research grant (Scotland Grant) from the Heart Research UK (RG2676/18/21). P. M. is supported by the British Heart Foundation (grants PG/19/84/34771, FS/19/56/34893A, PG/21/10541, PG/21/10557, PG/21/10634, PG/23/11680 and PG/24/11946), the University of Glasgow and the International Science Partnership Fund, FRA 2020–Linea A, University of Naples Federico II/Compagnia di San Paolo and the Italian Ministry of University and Research (MUR) PRIN 2022 (2022T45AXH). P. M. and A. Ialenti are supported by the European Union–Next Generation EU, Project CN00000041, Mission 4, Component 2, CUP B93D21010860004. T. J. G. is supported by the European Research Council (ERC and InflammaTENSION, ERC‐CoG‐726318); European Research Area–CVD (ERA‐CVD) [BrainGutImmune (ERA‐CVD/Gut‐brain/8/2021 and ImmmuneHyperCog, NCBiR Poland], British Heart Foundation grants (FS/14/49/30838 and FS/4yPhD/F/20/34127A) and as part of the British Heart Foundation Centre for Research Excellence at the University of Edinburgh (RE/18/5/34216). A. Ialenti is supported by the Italian Ministry of University and Research (MIUR) PRIN 2017 (2017NKB2N4_003). A. Iaconelli is supported by a grant from Fondazione Enrico ed Enrica Sovena (Rome, Italy).

## Supporting information


**Figure S1.** Associations of Neutrophil Count, Lymphocyte Count, and NLR with Survival according to LVEF phenotype: HFrEF. Kaplan Meier curves illustrating time to all‐cause mortality by quartile of neutrophil count (left panel), lymphocyte count (middle panel) and NLR (right panel). Unadjusted hazard ratios (HR) and 95% confidence intervals (95% CI) for all‐cause mortality are presented per unit change of white cell variable.

**Figure S2.** Associations of Neutrophil Count, Lymphocyte Count, and NLR with Survival according to LVEF phenotype: HFmrEF. Kaplan Meier curves illustrating time to all‐cause mortality by quartile of neutrophil count (left panel), lymphocyte count (middle panel) and NLR (right panel). Unadjusted hazard ratios (HR) and 95% confidence intervals (95% CI) for all‐cause mortality are presented per unit change of white cell variable.

**Figure S3.** Associations of Neutrophil Count, Lymphocyte Count, and NLR with Survival according to LVEF phenotype: HFpEF. Kaplan Meier curves illustrating time to all‐cause mortality by quartile of neutrophil count (left panel), lymphocyte count (middle panel) and NLR (right panel). Unadjusted hazard ratios (HR) and 95% confidence intervals (95% CI) for all‐cause mortality are presented per unit change of white cell variable.

**Table S1.** Baseline characteristics of patients with heart failure stratified by HF phenotype.

**Table S2.** Baseline characteristics of patients with heart failure stratified by quartiles of neutrophil count.

**Table S3.** Baseline characteristics of patients with heart failure stratified by quartiles of lymphocyte count.

**Table S4.** Baseline characteristics of patients with heart failure stratified by HF phenotype in a subset of patients with detailed echocardiography.

**Table S5.**Baseline characteristics of patients with heart failure stratified by quartiles of neutrophil‐to‐lymphocyte ratio in a subset of patients with detailed echocardiography.

**Table S6.** Rate, cause, and mode of death at 2 years by quartile of neutrophil count according to different HF phenotypes.

**Table S7.** Rate, cause, and mode of death at 2 years by quartile of lymphocyte count according to different HF phenotypes.

**Table S8.** Rate, cause, and mode of death at 2 years by quartile of NLR according to different HF phenotypes.
