# Supplementary figures and images for: Neutrophil‐to‐lymphocyte ratio: link to congestion, inflammation, and mortality in outpatients with heart failure
Source: ESC Heart Fail. 2025 Mar 2;12(3):1571–82. doi: 10.1002/ehf2.15240 (PMC12055385; doi:10.1002/ehf2.15240)

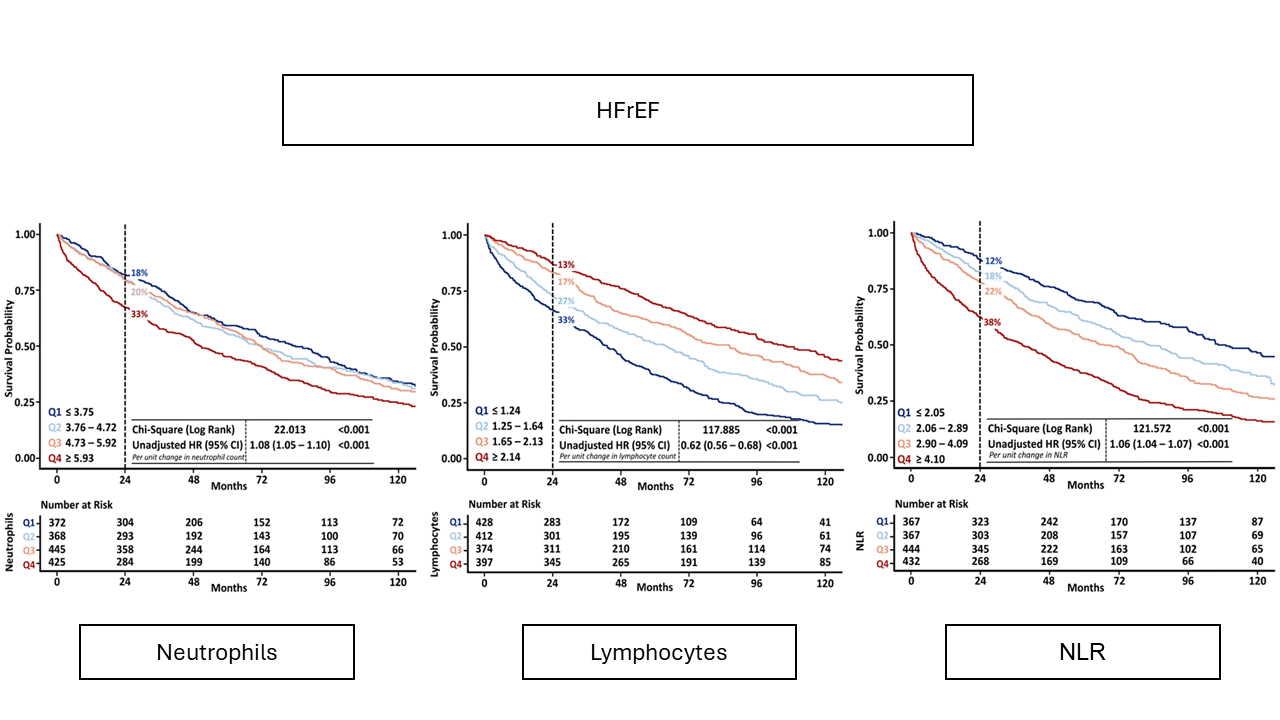

Supplement: Supplementary file 1 — Figure S1. Associations of Neutrophil Count, Lymphocyte Count, and NLR with Survival according to LVEF phenotype: HFrEF. Kaplan Meier curves illustrating time to all‐cause mortality by quartile of neutrophil count (left panel), lymphocyte count (middle panel) and NLR (right panel). Unadjusted hazard ratios (HR) and 95% confidence intervals (95% CI) for all‐cause mortality are presented per unit change of white cell variable. [file EHF2-12-1571-s001.tif]

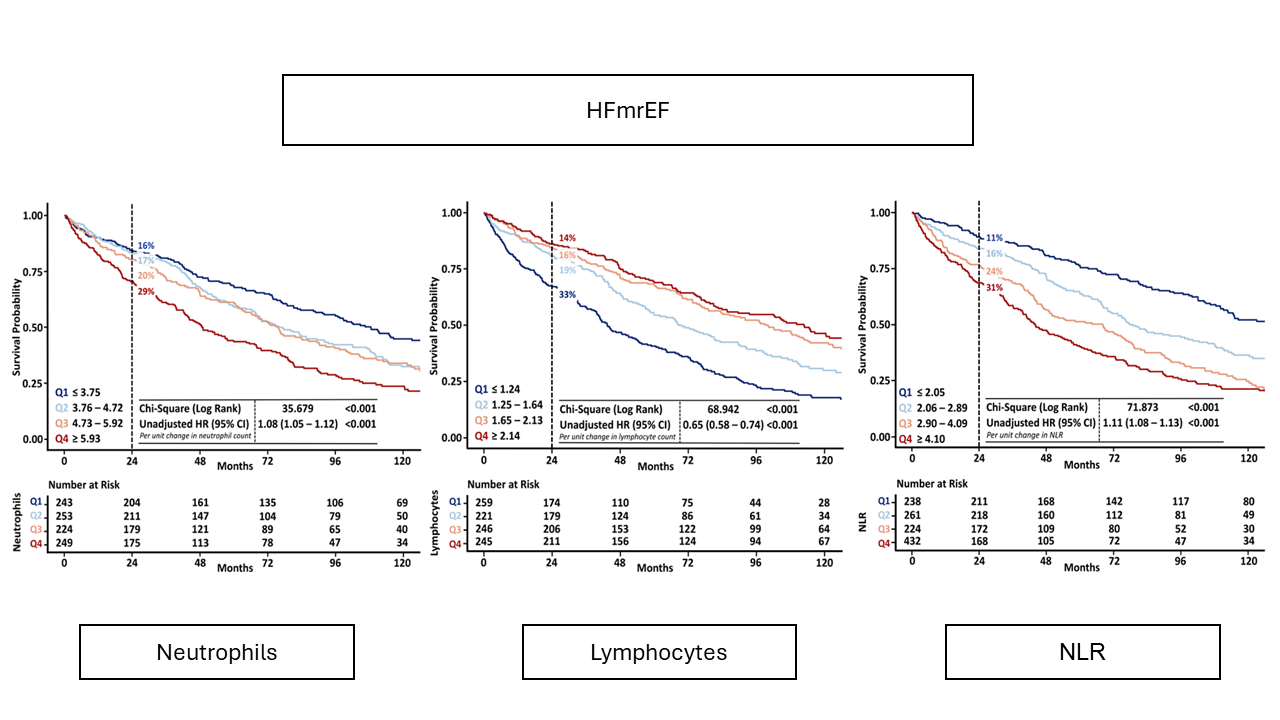

Supplement: Supplementary file 2 — Figure S2. Associations of Neutrophil Count, Lymphocyte Count, and NLR with Survival according to LVEF phenotype: HFmrEF. Kaplan Meier curves illustrating time to all‐cause mortality by quartile of neutrophil count (left panel), lymphocyte count (middle panel) and NLR (right panel). Unadjusted hazard ratios (HR) and 95% confidence intervals (95% CI) for all‐cause mortality are presented per unit change of white cell variable. [file EHF2-12-1571-s002.tif]

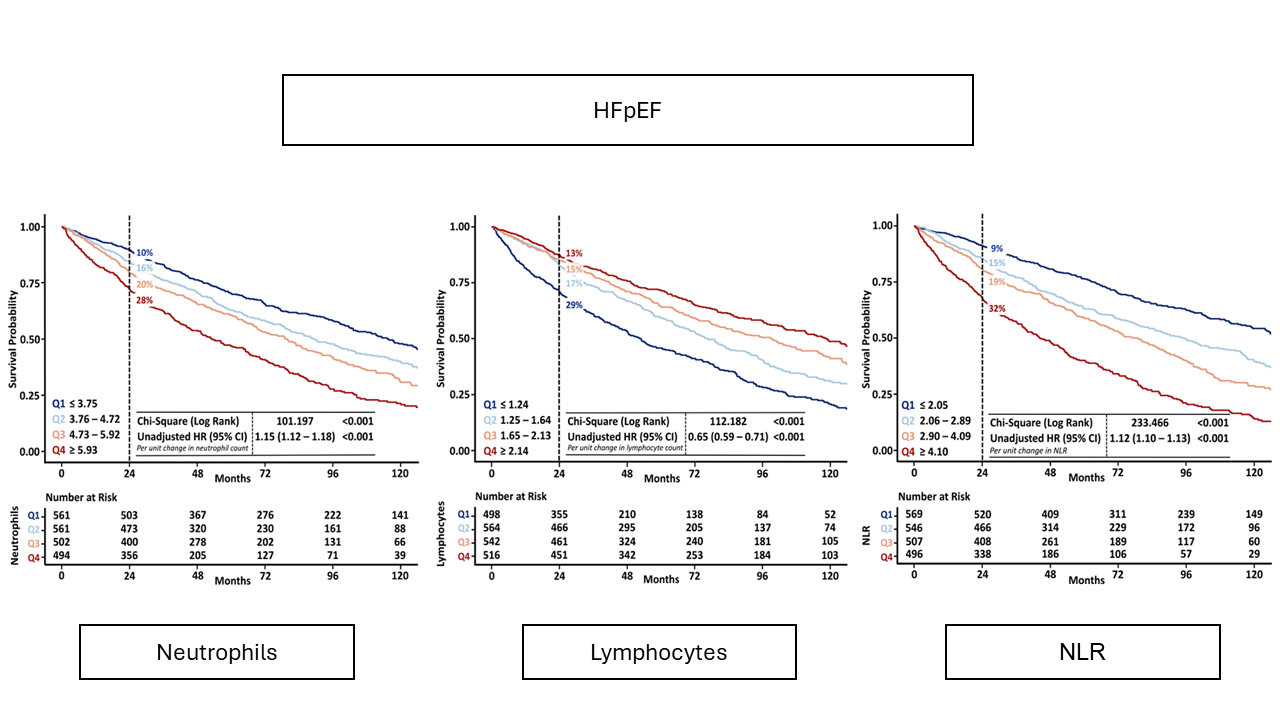

Supplement: Supplementary file 3 — Figure S3. Associations of Neutrophil Count, Lymphocyte Count, and NLR with Survival according to LVEF phenotype: HFpEF. Kaplan Meier curves illustrating time to all‐cause mortality by quartile of neutrophil count (left panel), lymphocyte count (middle panel) and NLR (right panel). Unadjusted hazard ratios (HR) and 95% confidence intervals (95% CI) for all‐cause mortality are presented per unit change of white cell variable. [file EHF2-12-1571-s009.tif]
