# Supplementary material for: Neutrophil‐to‐lymphocyte ratio: link to congestion, inflammation, and mortality in outpatients with heart failure
Source: ESC Heart Fail. 2025 Mar 2;12(3):1571–82. doi: 10.1002/ehf2.15240 (PMC12055385; doi:10.1002/ehf2.15240)
Supplement: Supplementary file 4 — Table S1. Baseline characteristics of patients with heart failure stratified by HF phenotype. [file EHF2-12-1571-s004.docx]

| **Variable** | | **Missing*** | **All**  **N=4702** | **HFrEF**  **N=1611** | **HFmrEF**  **N=971** | **HFpEF**  **N=2120** | **P** |
| --- | --- | --- | --- | --- | --- | --- | --- |
| **Demographics** | | | | | | | |
| **Age (years)** | | 0 (0) | 76 (68 – 82) | 73 (64 – 80) | 75 (68 – 81) | 78 (71 – 83) | **<0.001** |
| **Sex (women)** | | 0 (0) | 1913 (41) | 445 (28) | 315 (32) | 1153 (54) | **<0.001** |
| **Diabetes, n. (%)** | | 0 (0) | 1135 (24) | 351 (22) | 242 (25) | 542 (26) | **0.023** |
| **Hypertension, n. (%)** | | 0 (0) | 2196 (47) | 534 (33) | 436 (45) | 1226 (58) | **<0.001** |
| **IHD, n. (%)** | | 0 (0) | 1900 (40) | 855 (53) | 512 (53) | 533 (25) | **<0.001** |
| **COPD, n. (%)** | | 0 (0) | 454 (10) | 162 (10) | 87 (9) | 205 (10) | 0.659 |
| **BMI (kg/m^2^)** | | 18 (<1) | 28.1 (24.6 – 32.2) | 27.1 (23.8 – 30.7) | 28.5 (25.0 – 32.6) | 28.8 (25.1 – 32.9) | **<0.001** |
| **Systolic BP (mmHg)** | | 6 (<1) | 139 (122 – 158) | 128 (114 – 146) | 139 (122 – 158) | 148 (131 – 165) | **<0.001** |
| **Diastolic BP (mmHg)** | | 5 (<1) | 78 (69 – 88) | 76 (67 – 86) | 78 (69 – 89) | 79 (70 – 89) | **<0.001** |
| **Clinical Examination – Symptoms & Signs** | | | | | | | |
| **Peripheral Oedema ≥Ankles, n. (%)** | | 229 (5) | 459 (10) | 143 (9) | 87 (10) | 229 (11) | 0.160 |
| **Lung Crackles, n. (%)** | | 419 (9) | 617 (14) | 257 (18) | 133 (15) | 227 (12) | **<0.001** |
| **Raised JVP, n. (%)** | | 474 (10) | 701 (17) | 315 (22) | 133 (16) | 253 (13) | **<0.001** |
| **Liver Distension, n. (%)** | | 1750 (37) | 125 (3) | 61 (6) | 25 (4) | 39 (3) | **0.004** |
| **NYHA III/IV, n. (%)** | | 0 (0) | 1339 (28) | 590 (37) | 271 (28) | 478 (23) | **<0.001** |
| **ECG** | | | | | | | |
| **Heart Rate (bpm)** | | 4 (<1) | 74 (63 – 86) | 77 (66 – 91) | 72 (62 – 86) | 72 (62 – 83) | **<0.001** |
| **Atrial Fibrillation, n. (%)** | | 62 (1) | 1666 (36) | 481 (31) | 362 (38) | 823 (39) | **<0.001** |
| **QRS Width (msec)** | | 169 (4) | 100 (88 – 122) | 114 (98 – 144) | 102 (90 – 122) | 92 (82 – 104) | **<0.001** |
| **Echocardiography** | | | | | | | |
| **LVEDD (cm)** | | 743 (16) | 5.2 (4.7 – 5.9) | 6.1 (5.5 – 6.6) | 5.3 (4.9 – 5.8) | 4.8 (4.4 – 5.2) | **<0.001** |
| **Left Atrial Dimeter (cm)** | | 700 (15) | 4.1 (3.7 – 4.6) | 4.3 (3.9 – 4.8) | 4.2 (3.7 – 4.7) | 4.0 (3.6 – 4.5) | **<0.001** |
| **Mitral Regurgitation ≥ Mild** | | 648 (14) | 2836 (70) | 1183 (84) | 587 (69) | 1066 (59) | **<0.001** |
| **Blood Tests** | | | | | | | |
| **NTproBNP (ng/L)** | **Overall** | 513 (11) | 1145 (459 – 2588) | 2003 (905 – 4391) | 1160 (474 – 2623) | 806 (321 – 1752) | **<0.001** |
|  | **SR** |  | 722 (299 – 1824) | 1635 (723 – 3792) | 750 (306 – 1803) | 409 (227 – 905) | **<0.001** |
|  | **AF** |  | 1954 (1106 – 3425) | 2982 (1654 – 5714) | 2008 (1066 – 3717) | 1590 (1004 – 2614) | **<0.001** |
| **Serum Creatinine (µmol/L)** | | 223 (5) | 98 (80 – 122) | 104 (86 – 129) | 98 (81 – 123) | 92 (76 – 115) | **<0.001** |
| **eGFR (mL/min/1.73 m^2^)** | | 223 (5) | 59 (44 – 73) | 58 (44 – 71) | 59 (44 – 73) | 59 (46 – 74) | 0.067 |
| **Urea (mmol/L)** | | 124 (3) | 7.1 (5.4 – 9.7) | 7.6 (5.7 – 10.4) | 6.9 (5.4 – 9.4) | 6.9 (5.3 – 9.3) | **<0.001** |
| **Albumin (g/L)** | | 344 (7) | 37 (35 – 40) | 38 (35 – 40) | 37 (35 – 39) | 37 (35 – 40) | 0.72 |
| **Haemoglobin (g/dL)** | **All** | 123 (3) | 13.5 (12.3 – 14.7) | 13.3 (12.0 – 14.4) | 13.0 (11.8 – 14.2) | 13.4 (12.1 – 14.6) | **<0.001** |
|  | ***Women*** |  | 12.8 (11.9 – 13.8) | 12.7 (11.8 – 13.7) | 12.7 (11.7 – 13.7) | 12.7 (11.8 – 13.6) | 0.084 |
|  | ***Men*** |  | 13.8 (12.5 – 15.0) | 13.7 (12.3 – 14.8) | 13.5 (12.1 – 14.6) | 13.9 \|(12.4 – 14.9) | **<0.001** |
| **WBC Count (x10^9^/L)** | | 0 (0) | 7.4 (6.2 – 8.8) | 7.6 (6.3 – 9.0) | 7.4 (6.1 – 8.9) | 7.3 (6.1 – 8.7) | **0.001** |
| **Neutrophil Count (x10^9^/L)** | | 5 (<1) | 4.72 (3.75 – 5.92) | 4.90 (3.83 – 6.04) | 4.67 (3.74 – 5.96) | 4.62 (3.69 – 5.81) | **<0.001** |
| **Lymphocyte Count (x10^9^/L)** | | 0 (0) | 1.64 (1.24 – 2.13) | 1.60 (1.21 – 2.12) | 1.65 (1.21 – 2.14) | 1.64 (1.27 – 2.12) | 0.13 |
| **Neutrophil-to-Lymphocyte Ratio** | | 5 (<1) | 2.89 (2.05 – 4.09) | 3.01 (2.13 – 4.23) | 2.81 (2.06 – 4.11) | 2.77 (2.01 – 3.98) | **<0.001** |
| **Monocyte Count (x10^9^/L)** | | 1 (<1) | 0.64 (0.51 – 0.80) | 0.65 (0.52 – 0.81) | 0.66 (0.52 – 0.83) | 0.63 (0.50 – 0.78) | **<0.001** |
| **Eosinophil Count (x10^9^/L)** | | 86 (2) | 0.16 (0.10 – 0.25) | 0.16 (0.10 – 0.26) | 0.17 (0.10 – 0.27) | 0.15 (0.09 – 0.24) | **0.026** |
| **Basophil Count (x10^9^/L)** | | 176 (4) | 0.03 (0.02 – 0.04) | 0.03 (0.02 – 0.04) | 0.03 (0.02 – 0.04) | 0.00 (0.02 – 0.04) | 0.48 |
| **hsCRP (mg/L)** | | 908 (19) | 4.1 (1.7 – 9.0) | 4.4 (1.7 – 9.9) | 4.3 (1.7 – 8.8) | 4.0 (1.7 – 8.8) | 0.30 |
| **Treatment at Time of Referral** | | | | | | | |
| **Loop Diuretic, n. (%)** | | 0 (0) | 2929 (62) | 1226 (76) | 615 (63) | 1088 (51) | **<0.001** |
| **>40 mg Furosemide/day, n. (%)** | | 0 (0) | 1164 (25) | 586 (36) | 227 (23) | 351 (16) | **<0.001** |
| **Beta Blocker, n. (%)** | | 0 (0) | 2755 (59) | 1016 (63) | 638 (66) | 1101 (52) | **<0.001** |
| **ACEi, n. (%)** | | 0 (0) | 2615 (56) | 1117 (69) | 599 (62) | 899 (42) | **<0.001** |
| **ARB, n. (%)** | | 0 (0) | 573 (12) | 145 (9) | 108 (11) | 320 (15) | **<0.001** |
| **MRA, n. (%)** | | 0 (0) | 867 (18) | 518 (32) | 182 (19) | 167 (8) | **<0.001** |

**Supplementary Table 1.** **Baseline characteristics of patients with heart failure stratified by HF phenotype.**

Abbreviations used: HF, heart failure; IHD, ischaemic heart disease; COPD, chronic obstructive pulmonary disease; BMI, body mass index; BP, blood pressure; JVP, jugular vein pressure; NYHA, New York Heart Association; HFrEF, heart failure with reduced ejection fraction; HFmrEF, heart failure with mildly reduced ejection fraction; HFpEF, heart failure with preserved ejection fraction; LVEDD, left ventricular end-diastolic diameter; NTproBNP, N-terminal pro–B-type natriuretic peptide; SR, sinus rhythm; AF, atrial fibrillation; eGFR, estimated glomerular filtration rate; WBC, white blood cell; hsCRP, high sensitivity C-reactive protein; ACEi, angiotensin-converting enzyme inhibitor; ARB, angiotensin receptor blocker; MRA, mineralocorticoid receptor antagonist. *Missing refers to missing values from the overall included patients, n = 4702.
