# Supplementary material for: Neutrophil‐to‐lymphocyte ratio: link to congestion, inflammation, and mortality in outpatients with heart failure
Source: ESC Heart Fail. 2025 Mar 2;12(3):1571–82. doi: 10.1002/ehf2.15240 (PMC12055385; doi:10.1002/ehf2.15240)
Supplement: Supplementary file 5 — Table S2. Baseline characteristics of patients with heart failure stratified by quartiles of neutrophil count. [file EHF2-12-1571-s006.docx]

| **Variable** | | **Missing*** | **Decile 1**  **≤ 3.04**  **N = 469** | **Quartile 1**  **≤ 3.75**  **N= 1176** | **Quartile 2**  **3.76 – 4.72**  **N= 1182** | **Quartile 3**  **4.73 – 5.92**  **N= 1171** | **Quartile 4**  **≥ 5.93**  **N= 1168** | **Decile 10**  **≥ 7.49**  **N= 470** | **P** |
| --- | --- | --- | --- | --- | --- | --- | --- | --- | --- |
| **Demographics** | | | | | | | | | |
| **Age (years)** | | 0 (0) | 74 (67 – 80) | 75 (67 – 81) | 76 (69 – 82) | 75 (68 – 82) | 76 (69 – 82) | 76 (69 – 82) | 0.12 |
| **Sex (women)** | | 0 (0) | 212 (45) | 486 (41) | 498 (42) | 449 (38) | 477 (41) | 198 (42) | 0.27 |
| **Diabetes, n. (%)** | | 0 (0) | 74 (16) | 200 (17) | 270 (23) | 300 (26) | 364 (31) | 157 (33) | **<0.001** |
| **Hypertension, n. (%)** | | 0 (0) | 223 (48) | 546 (46) | 563 (48) | 555 (47) | 531 (46) | 199 (42) | 0.71 |
| **IHD, n. (%)** | | 0 (0) | 178 (38) | 468 (40) | 479 (40) | 466 (40) | 484 (41) | 193 (41) | 0.83 |
| **COPD, n. (%)** | | 0 (0) | 28 (6) | 79 (7) | 105 (9) | 117 (10) | 153 (13) | 70 (15) | **<0.001** |
| **BMI (kg/m^2^)** | | 18 (<1) | 27.5 (24.3 – 31.4) | 27.6 (24.3 – 31.3) | 28.3 (24.7 – 32.3) | 28.5 (25.0 – 32.6) | 28.1 (24.3 – 32.6) | 27.9 (24.3 – 32.3) | **<0.001** |
| **Systolic BP (mmHg)** | | 6 (<1) | 140 (124 – 159) | 140 (123 – 159) | 140 (122 – 158) | 140 (122 – 159) | 138 (120 – 157) | 134 (117 – 153) | 0.094 |
| **Diastolic BP (mmHg)** | | 5 (<1) | 79 (69 – 89) | 78 (69 – 88) | 78 (70 – 88) | 78 (69 – 89) | 77 (67 – 87) | 75 (64 – 85) | **0.005** |
| **Clinical Examination – Symptoms & Signs** | | | | | | | | | |
| **Peripheral Oedema ≥Ankles, n. (%)** | | 229 (5) | 31 (7) | 80 (7) | 108 (10) | 125 (11) | 145 (13) | 68 (16) | **<0.001** |
| **Lung Crackles, n. (%)** | | 419 (9) | 35 (8) | 110 (10) | 135 (13) | 164 (16) | 206 (19) | 101 (24) | **<0.001** |
| **Raised JVP, n. (%)** | | 474 (10) | 64 (15) | 146 (14) | 166 (16) | 179 (17) | 210 (20) | 101 (24) | **0.001** |
| **Liver Distension, n. (%)** | | 1750 (37) | 11 (4) | 28 (4) | 21 (3) | 33 (5) | 43 (6) | 28 (10) | **0.035** |
| **NYHA III/IV, n. (%)** | | 0 (0) | 83 (18) | 238 (20) | 311 (26) | 351 (30) | 437 (37) | 205 (44) | **<0.001** |
| **ECG** | | | | | | | | | |
| **Heart Rate (bpm)** | | 4 (<1) | 69 (60 – 80) | 70 (60 – 81) | 72 (62 – 84) | 75 (64 – 87) | 79 (67 – 92) | 81 (70 – 96) | **<0.001** |
| **Atrial Fibrillation, n. (%)** | | 62 (1) | 151 (32) | 372 (32) | 416 (36) | 445 (38) | 431 (37) | 173 (37) | **0.008** |
| **QRS Width (msec)** | | 169 (4) | 98 (88 – 120) | 99 (88 – 122) | 100 (88 – 118) | 100 (88 – 126) | 98 (86 – 120) | 98 (86 – 120) | **0.019** |
| **Echocardiography** | | | | | | | | | |
| **HF phenotype** | **HFrEF** | 0 (0) | 132 (28) | 372 (32) | 368 (31) | 445 (38) | 425 (36) | 179 (38) | **0.002** |
|  | **HFmrEF** |  | 98 (21) | 243 (21) | 253 (21) | 224 (19) | 249 (21) | 101 (22) |  |
|  | **HFpEF** |  | 239 (51) | 561 (48) | 561 (48) | 502 (43) | 494 (42) | 190 (40) |  |
| **LVEDD (cm)** | | 743 (16) | 5.2 (4.6 – 5.9) | 5.2 (4.6 – 6.0) | 5.2 (4.6 – 5.8) | 5.3 (4.7 – 6.0) | 5.2 (4.6 – 5.9) | 5.2 (4.6 – 5.9) | 0.15 |
| **Left Atrial Dimeter (cm)** | | 700 (15) | 4.1 (3.6 – 4.6) | 4.1 (3.6 – 4.6) | 4.2 (3.7 – 4.7) | 4.2 (3.8 – 4.7) | 4.1 (3.7 – 4.6) | 4.1 (3.7 – 4.7) | 0.088 |
| **Mitral Regurgitation ≥ Mild** | | 648 (14) | 281 (67) | 722 (69) | 724 (69) | 723 (72) | 665 (70) | 262 (68) | 0.45 |
| **Blood Tests** | | | | | | | | | |
| **NTproBNP (ng/L)** | **Overall** | 513 (11) | 812 (305 – 1880) | 893 (330 – 2098) | 1131 (458 – 2380) | 1304 (534 – 2816) | 1379 (606 – 3185) | 1459 (636 – 3369) | **<0.001** |
|  | **SR** |  | 473 (220 – 1136) | 539 (250 – 1283) | 737 (319 – 1756) | 763 (316 – 2146) | 830 (374 – 2368) | 982 (478 – 2614) | **<0.001** |
|  | **AF** |  | 1690 (926 – 3204) | 1857 (1039 – 32.85) | 1804 (1051 – 3190) | 1996 (1164 – 3360) | 2248 (1207 – 4017) | 2154 (1151 – 4016) | **0.006** |
| **Serum Creatinine (µmol/L)** | | 223 (5) | 91 (75 – 110) | 94 (78 – 113) | 96 (80 – 120) | 99 (81 – 123) | 102 (81 – 134) | 104 (80 – 138) | **<0.001** |
| **eGFR (mL/min/1.73 m^2^)** | | 223 (5) | 63 (50 – 77) | 62 (49 – 75) | 59 (46 – 73) | 59 (44 – 73) | 55 (40 – 71) | 54 (39 – 71) | **<0.001** |
| **Urea (mmol/L)** | | 124 (3) | 6.4 (5.0 – 8.2) | 6.5 (5.1 – 8.6) | 7.0 (5.5 – 9.5) | 7.1 (5.4 – 9.8) | 8.0 (5.9 – 11.3) | 8.4 (6.2 – 12.3) | **<0.001** |
| **Albumin (g/L)** | | 344 (7) | 38 (36 – 40) | 38 (36 – 40) | 38 (36 – 40) | 38 (35 – 40) | 36 (34 – 39) | 36 (33 – 38) | **<0.001** |
| **Haemoglobin (g/dL)** | **All** | 123 (3) | 13.2 (11.8 – 14.3) | 13.2 (12.0 – 14.4) | 13.3 (12.0 – 14.4) | 13.4 (12.2 – 14.6) | 13.1 (11.8 – 14.4) | 13.0 (11.6 – 14.2) | **0.001** |
|  | ***Women*** |  | 12.7 (11.7 – 13.7) | 12.7 (11.8 – 13.7) | 12.7 (11.7 – 13.6) | 12.8 (11.9 – 13.8) | 12.7 (11.6 – 13.7) | 12.6 (11.2 – 13.6) | 0.39 |
|  | ***Men*** |  | 13.6 (12.2 – 14.8) | 13.7 (12.3 – 14.7) | 13.7 (12.5 – 14.9) | 13.8 (12.5 – 14.9) | 13.6 (12.0 – 14.7) | 13.3 (11.9 – 14.5) | **0.006** |
| **WBC Count (x10^9^/L)** | | 0 (0) | 4.9 (4.4 – 5.4) | 5.5 (4.9 – 6.1) | 6.8 (6.2 – 7.4) | 7.9 (7.3 – 8.6) | 10.1 (9.0 – 11.6) | 11.8 (10.7 – 13.1) | **<0.005** |
| **Neutrophil Count (x10^9^/L)** | | 5 (<1) | 2.68 (2.41 – 2.87) | 3.18 (2.78 – 3.49) | 4.22 (3.98 – 4.48) | 5.23 (4.98 – 5.55) | 7.11 (6.43 – 8.37) | 8.74 (8.05 – 10.11) | - |
| **Lymphocyte Count (x10^9^/L)** | | 0 (0) | 1.51 (1.18 – 1.98) | 1.57 (1.19 – 2.05) | 1.64 (1.26 – 2.13) | 1.69 (1.30 – 2.18) | 1.64 (1.19 – 2.15) | 1.56 (1.12 – 2.08) | **<0.001** |
| **Monocyte Count (x10^9^/L)** | | 1 (<1) | 0.48 (0.39 – 0.60) | 0.51 (0.41 – 0.64) | 0.61 (0.50 – 0.73) | 0.68 (0.56 – 0.82) | 0.80 (0.64 – 0.98) | 0.87 (0.66 – 1.1) | **<0.001** |
| **Eosinophil Count (x10^9^/L)** | | 86 (2) | 0.14 (0.09 – 0.21) | 0.15 (0.09 – 0.23) | 0.17 (0.10 – 0.26) | 0.17 (0.10 – 0.27) | 0.15 (0.08 – 0.27) | 0.13 (0.06 – 0.24) | **<0.001** |
| **Basophil Count (x10^9^/L)** | | 176 (4) | 0.020 (0.020 – 0.040) | 0.030 (0.020 – 0.040) | 0.030 (0.020 – 0.040) | 0.030 (0.020 – 0.050) | 0.030 (0.020 – 0.050) | 0.030 (0.020 –0.050) | **<0.001** |
| **hsCRP (mg/L)** | | 908 (19) | 2.4 (1.1 – 5.6) | 2.6 (1.2 – 5.7) | 3.4 (1.5 – 7.2) | 4.5 (1.9 – 9.4) | 7.5 (3.3 – 18.0) | 9.6 (3.8 – 25.0) | **<0.001** |
| **Treatment at Time of Referral** | | | | | | | | | |
| **Loop Diuretic, n. (%)** | | 0 (0) | 241 (51) | 635 (54) | 712 (60) | 751 (64) | 829 (71) | 338 (72) | **<0.001** |
| **>40 mg Furosemide/day, n. (%)** | | 0 (0) | 62 (13) | 206 (18) | 271 (23) | 309 (26) | 377 (32) | 174 (37) | **<0.001** |
| **Beta Blocker, n. (%)** | | 0 (0) | 258 (55) | 687 (58) | 732 (62) | 692 (59) | 639 (55) | 232 (49) | **0.005** |
| **ACEi, n. (%)** | | 0 (0) | 236 (50) | 639 (54) | 665 (56) | 648 (55) | 660 (56) | 276 (59) | 0.71 |
| **ARB, n. (%)** | | 0 (0) | 62 (13) | 137 (12) | 146 (12) | 150 (13) | 140 (12) | 49 (10) | 0.85 |
| **MRA, n. (%)** | | 0 (0) | 65 (14) | 184 (16) | 186 (16) | 241 (21) | 254 (22) | 96 (20) | **<0.001** |

**Supplementary Table 2.** **Baseline characteristics of patients with heart failure stratified by quartiles of neutrophil count**; first and last deciles are also shown. Abbreviations used: HF, heart failure; IHD, ischaemic heart disease; COPD, chronic obstructive pulmonary disease; BMI, body mass index; BP, blood pressure; JVP, jugular vein pressure; NYHA, New York Heart Association; HFrEF, heart failure with reduced ejection fraction; HFmrEF, heart failure with mildly reduced ejection fraction; HFpEF, heart failure with preserved ejection fraction; LVEDD, left ventricular end-diastolic diameter; NTproBNP, N-terminal pro–B-type natriuretic peptide; SR, sinus rhythm; AF, atrial fibrillation; eGFR, estimated glomerular filtration rate; WBC, white blood cell; hsCRP, high sensitivity C-reactive protein; ACEi, angiotensin-converting enzyme inhibitor; ARB, angiotensin receptor blocker; MRA, mineralocorticoid receptor antagonist. *Missing refers to missing values from the overall included patients, n = 4702.
