# Supplementary material for: Neutrophil‐to‐lymphocyte ratio: link to congestion, inflammation, and mortality in outpatients with heart failure
Source: ESC Heart Fail. 2025 Mar 2;12(3):1571–82. doi: 10.1002/ehf2.15240 (PMC12055385; doi:10.1002/ehf2.15240)
Supplement: Supplementary file 6 — Table S3. Baseline characteristics of patients with heart failure stratified by quartiles of lymphocyte count. [file EHF2-12-1571-s008.docx]

| **Variable** | | **Missing*** | **Decile 1**  **≤ 0.93**  **N = 473** | **Quartile 1**  **≤ 1.24**  **N= 1185** | **Quartile 2**  **1.25 – 1.64**  **N= 1197** | **Quartile 3**  **1.65 – 2.13**  **N= 1162** | **Quartile 4**  **≥ 2.14**  **N= 1158** | **Decile 10**  **≥ 2.66**  **N= 468** | **P** |
| --- | --- | --- | --- | --- | --- | --- | --- | --- | --- |
| **Demographics** | | | | | | | | | |
| **Age (years)** | | 0 (0) | 79 (73 – 85) | 78 (72 – 83) | 77 (70 – 83) | 75 (67 – 82) | 72 (64 – 79) | 71 (62 – 79) | **<0.001** |
| **Sex (women)** | | 0 (0) | 166 (35) | 433 (36) | 488 (41) | 493 (42) | 499 (43) | 198 (42) | **0.005** |
| **Diabetes, n. (%)** | | 0 (0) | 97 (20) | 249 (21) | 304 (25) | 271 (23) | 311 (27) | 134 (29) | **0.006** |
| **Hypertension, n. (%)** | | 0 (0) | 203 (43) | 521 (44) | 581 (48) | 551 (47) | 543 (47) | 224 (48) | 0.14 |
| **IHD, n. (%)** | | 0 (0) | 198 (42) | 491 (41) | 481 (40) | 436 (38) | 492 (42) | 207 (44) | 0.084 |
| **COPD, n. (%)** | | 0 (0) | 52 (11) | 131 (11) | 105 (9) | 102 (9) | 116 (10) | 42 (9) | 0.18 |
| **BMI (kg/m^2^)** | | 18 (<1) | 26.0 (22.9 – 29.9) | 26.3 (23.4 – 30.4) | 28.0 (24.6 – 31.8) | 28.7 (25.1 – 32.4) | 29.2 (25.6 – 33,6) | 28.9 (25.6 – 34.0) | **<0.001** |
| **Systolic BP (mmHg)** | | 6 (<1) | 136 (114 – 156) | 138 (118 – 157) | 140 (124 – 159) | 140 (123 – 158) | 140 (122 – 160) | 141 (122 – 164) | **<0.003** |
| **Diastolic BP (mmHg)** | | 5 (<1) | 74 (64 – 85) | 76 (66 – 87) | 77 (68 – 87) | 78 (69 – 89) | 79 (70 – 89) | 80 (71 – 89) | **<0.001** |
| **Clinical Examination – Symptoms & Signs** | | | | | | | | | |
| **Peripheral Oedema ≥Ankles, n. (%)** | | 229 (5) | 99 (22) | 201 (18) | 120 (11) | 80 (7) | 58 (5) | 18 (4) | **<0.001** |
| **Lung Crackles, n. (%)** | | 419 (9) | 102 (24) | 221 (20) | 160 (15) | 117 (11) | 119 (11) | 39 (9) | **<0.001** |
| **Raised JVP, n. (%)** | | 474 (10) | 139 (34) | 273 (26) | 173 (16) | 148 (14) | 107 (10) | 42 (10) | **<0.001** |
| **Liver Distension, n. (%)** | | 1750 (37) | 32 (11) | 57 (7) | 31 (4) | 26 (4) | 11 (2) | 5 (2) | **<0.001** |
| **NYHA III/IV, n. (%)** | | 0 (0) | 211 (45) | 434 (37) | 362 (30) | 302 (26) | 241 (21) | 97 (21) | **<0.001** |
| **ECG** | | | | | | | | | |
| **Heart Rate (bpm)** | | 4 (<1) | 75 (64 – 88) | 74 (64 – 87) | 74 (63 – 87) | 73 (63 – 87) | 73 (63 – 85) | 72 (62 – 84) | 0.093 |
| **Atrial Fibrillation, n. (%)** | | 62 (1) | 217 (47) | 511 (44) | 432 (36) | 406 (35) | 317 (28) | 115 (25) | **<0.001** |
| **QRS Width (msec)** | | 169 (4) | 104 (90 – 132) | 102 (88 – 128) | 100 (88 – 126) | 98 (86 – 120) | 98 (88 – 116) | 98 (88 – 114) | **<0.001** |
| **Echocardiography** | | | | | | | | | |
| **HF phenotype** | **HFrEF** | 0 (0) | 185 (39) | 428 (36) | 412 (34) | 374 (32) | 397 (34) | 160 (34) | 0.094 |
|  | **HFmrEF** |  | 95 (20) | 259 (22) | 221 (18) | 246 (21) | 245 (21) | 102 (22) |  |
|  | **HFpEF** |  | 193 (41) | 498 (42) | 564 (47) | 542 (47) | 516 (45) | 206 (44) |  |
| **LVEDD (cm)** | | 743 (16) | 5.3 (4.6 – 6.0) | 5.2 (4.6 – 6.0) | 5.2 (4.7 – 5.8) | 5.2 (4.6 – 5.9) | 5.3 (4.7 – 6.0) | 5.3 (4.8 – 5.9) | 0.52 |
| **Left Atrial Dimeter (cm)** | | 700 (15) | 4.4 (3.8 – 5.0) | 4.3 (3.8 – 4.8) | 4.2 (3.7 – 4.6) | 4.1 (3.7 – 4.6) | 4.0 (3.6 – 4.5) | 4.0 (3.6 – 4.4) | **<0.001** |
| **Mitral Regurgitation ≥ Mild** | | 648 (14) | 331 (80) | 795 (77) | 748 (72) | 663 (67) | 630 (64) | 242 (60) | **<0.001** |
| **Blood Tests** | | | | | | | | | |
| **NTproBNP (ng/L)** | **Overall** | 513 (11) | 2552 (948 – 5498) | 1872 (755 – 4165) | 1246 (532 – 2643) | 1023 (398 – 2258) | 782 (333 – 1699) | 748 (280 – 1661) | **<0.001** |
|  | **SR** |  | 1381 (479 – 4232) | 1125 (418 – 3216) | 802 (324 – 1956) | 660 (264 – 1701) | 541 (248 – 1281) | 501 (232 – 1288) | **<0.001** |
|  | **AF** |  | 3578 (1796 – 6524) | 2662 (1429 – 5304) | 2008 (1139 – 3402) | 1768 (1012 – 2914) | 1489 (921 – 2296) | 1518 (974 – 2130) | **<0.001** |
| **Serum Creatinine (µmol/L)** | | 223 (5) | 108 (88 – 138) | 104 (83 – 130) | 100 (81 – 124) | 96 (79 – 118) | 93 (78 – 113) | 92 (77 – 110) | **<0.001** |
| **eGFR (mL/min/1.73 m^2^)** | | 223 (5) | 51 (39 – 66) | 55 (42 – 70) | 58 (43 – 71) | 60 (46 – 73) | 62 (48 – 77) | 64 (50 – 78) | **<0.001** |
| **Urea (mmol/L)** | | 124 (3) | 8.4 (6.4 – 11.5) | 7.7 (5.8 – 10.9) | 7.3 (5.6 – 10.2) | 6.8 (5.3 – 9.4) | 6.7 (5.1 – 8.7) | 6.4 (4.9 – 8.7) | **<0.001** |
| **Albumin (g/L)** | | 344 (7) | 36 (33 – 38) | 37 (34 – 39) | 37 (35 – 39) | 38 (35 – 40) | 38 (36 – 40) | 38 (36 – 40) | **<0.001** |
| **Haemoglobin (g/dL)** | **All** | 123 (3) | 12.2 (11.0 – 13.5) | 12.6 (11.3 – 13.9) | 13.0 (11.9 – 14.2) | 13.5 (12.4 – 14.6) | 13.7 (12.6 – 14.9) | 13.8 (12.7 – 15.0) | **<0.001** |
|  | ***Women*** |  | 12.0 (11.0 – 13.2) | 12.2 (11.1 – 13.4) | 12.6 (11.5 – 13.5) | 13.0 (12.1 – 13.9) | 13.0 (12.2 – 14.0) | 13.0 (12.2 – 13.9) | **<0.001** |
|  | ***Men*** |  | 12.3 (11.0 – 13.7) | 12.9 (11.4 – 14.2) | 13.5 (12.2 – 14.6) | 14.0 (12.7 – 15.0) | 14.4 (13.3 – 15.3) | 14.5 (13.3 – 15.4) | **<0.001** |
| **WBC Count (x10^9^/L)** | | 0 (0) | 6.4 (5.1 – 8.1) | 6.5 (5.4 – 7.9) | 7.0 (5.9 – 8.2) | 7.5 (6.5 – 8.8) | 8.5 (7.4 – 10.0) | 9.2 (8.1 – 10.6) | **<0.001** |
| **Neutrophil Count (x10^9^/L)** | | 5 (<1) | 4.76 (3.68 – 6.55) | 4.66 (3.65 – 6.10) | 4.65 (3.71 – 5.80) | 4.73 (3.79 – 5.88) | 4.87 (3.86 – 5.96) | 4.97 (4.06 – 6.24) | **0.030** |
| **Lymphocyte Count (x10^9^/L)** | | 0 (0) | 0.76 (0.64 – 0.86) | 0.99 (0.81 – 1.13) | 1.45 (1.35 – 1.54) | 1.86 (1.75 – 2.00) | 2.56 (2.31 – 2.91) | 3.00 (2.81 – 3.30) | - |
| **Monocyte Count (x10^9^/L)** | | 1 (<1) | 0.56 (0.42 – 0.72) | 0.58 (0.46 – 0.74) | 0.62 (0.50 – 0.77) | 0.65 (0.53 – 0.81) | 0.70 (0.57 – 0.87) | 0.77 (0.61 – 0.93) | **<0.001** |
| **Eosinophil Count (x10^9^/L)** | | 86 (2) | 0.11 (0.05 – 0.18) | 0.12 (0.07 – 0.20) | 0.15 (0.09 – 0.23) | 0.17 (0.11 – 0.27) | 0.19 (0.13 – 0.30) | 0.20 (0.13 – 0.32) | **<0.001** |
| **Basophil Count (x10^9^/L)** | | 176 (4) | 0.020 (0.010 – 0.030) | 0.030 (0.020 – 0.040) | 0.030 (0.020 – 0.040) | 0.030 (0.020 – 0.040) | 0.030 (0.020 – 0.050) | 0.040 (0.020 –0.050) | **<0.001** |
| **hsCRP (mg/L)** | | 908 (19) | 6.0 (2.4 – 18.0) | 5.3 (2.1 – 13.0) | 4.1 (1.5 – 9.6) | 3.7 (1.7 – 7.1) | 3.7 (1.6 – 7.8) | 4.0 (1.6 – 8.5) | **<0.001** |
| **Treatment at Time of Referral** | | | | | | | | | |
| **Loop Diuretic, n. (%)** | | 0 (0) | 339 (72) | 785 (66) | 769 (64) | 704 (61) | 671 (58) | 280 (60) | **<0.001** |
| **>40 mg Furosemide/day, n. (%)** | | 0 (0) | 161 (34) | 345 (29) | 325 (27) | 249 (21) | 245 (21) | 101 (22) | **<0.001** |
| **Beta Blocker, n. (%)** | | 0 (0) | 245 (52) | 632 (53) | 685 (57) | 701 (60) | 737 (64) | 297 (64) | **<0.001** |
| **ACEi, n. (%)** | | 0 (0) | 252 (53) | 636 (54) | 662 (55) | 640 (55) | 677 (58) | 283 (60) | 0.12 |
| **ARB, n. (%)** | | 0 (0) | 54 (11) | 122 (10) | 149 (12) | 144 (12) | 158 (14) | 55 (12) | 0.094 |
| **MRA, n. (%)** | | 0 (0) | 86 (18) | 213 (18) | 211 (18) | 195 (17) | 248 (21) | 99 (21) | **0.022** |

**Supplementary Table 3.** **Baseline characteristics of patients with heart failure stratified by quartiles of lymphocyte count**; first and last deciles are also shown. Abbreviations used: HF, heart failure; IHD, ischaemic heart disease; COPD, chronic obstructive pulmonary disease; BMI, body mass index; BP, blood pressure; JVP, jugular vein pressure; NYHA, New York Heart Association; HFrEF, heart failure with reduced ejection fraction; HFmrEF, heart failure with mildly reduced ejection fraction; HFpEF, heart failure with preserved ejection fraction; LVEDD, left ventricular end-diastolic diameter; NTproBNP, N-terminal pro–B-type natriuretic peptide; SR, sinus rhythm; AF, atrial fibrillation; eGFR, estimated glomerular filtration rate; WBC, white blood cell; hsCRP, high sensitivity C-reactive protein; ACEi, angiotensin-converting enzyme inhibitor; ARB, angiotensin receptor blocker; MRA, mineralocorticoid receptor antagonist. *Missing refers to missing values from the overall included patients, n = 4702.
