# Supplementary material for: Neutrophil‐to‐lymphocyte ratio: link to congestion, inflammation, and mortality in outpatients with heart failure
Source: ESC Heart Fail. 2025 Mar 2;12(3):1571–82. doi: 10.1002/ehf2.15240 (PMC12055385; doi:10.1002/ehf2.15240)
Supplement: Supplementary file 7 — Table S4. Baseline characteristics of patients with heart failure stratified by HF phenotype in a subset of patients with detailed echocardiography. [file EHF2-12-1571-s010.docx]

| **Variable** | | | **Missing*** | **All**  **N=813** | **HFrEF**  **N=341** | **HFmrEF**  **N=175** | **HFpEF**  **N=297** | **P** |
| --- | --- | --- | --- | --- | --- | --- | --- | --- |
| **Demographics** | | | | | | | | |
| **Age (years)** | | | 0 (0) | 75 (66 – 81) | 73 (65 – 79) | 72 (62 – 80) | 78 (72 – 84) | **<0.001** |
| **Sex (women)** | | | 0 (0) | 253 (31) | 87 (26) | 36 (21) | 130 (44) | **<0.001** |
| **Diabetes, n. (%)** | | | 0 (0) | 243 (30) | 100 (29) | 52 (30) | 91 (31) | 0.94 |
| **Hypertension, n. (%)** | | | 0 (0) | 445 (55) | 153 (45) | 87 (50) | 205 (69) | **<0.001** |
| **IHD, n. (%)** | | | 0 (0) | 498 (61) | 245 (72) | 118 (67) | 135 (46) | **<0.001** |
| **COPD, n. (%)** | | | 0 (0) | 96 (12) | 44 (13) | 17 (10) | 35 (12) | 0.57 |
| **BMI (kg/m^2^)** | | | 0 (0) | 28.5 (24.9 – 32.4) | 27.7 (24.2 – 31.0) | 29.5 (26.1 – 33.4) | 29.1 (25.1 – 33.6) | **<0.001** |
| **Systolic BP (mmHg)** | | | 0 (0) | 126 (112 – 143) | 121 (106 – 138) | 124 (113 – 139) | 134 (120 – 156) | **<0.001** |
| **Clinical Examination – Symptoms & Signs** | | | | | | | | |
| **Peripheral Oedema ≥Moderate, n. (%)** | | | 0 (0) | 162 (20) | 54 (16) | 32 (18) | 76 (26) | **0.007** |
| **Lung Crackles, n. (%)** | | | 0 (0) | 106 (13) | 46 (14) | 22 (13) | 38 (13) | 0.95 |
| **Raised JVP, n. (%)** | | | 0 (0) | 86 (11) | 38 (11) | 16 (9) | 32 (11) | 0.78 |
| **Liver Distension, n. (%)** | | | 0 (0) | 39 (5) | 17 (5) | 5 (3) | 17 (6) | 0.36 |
| **NYHA III/IV, n. (%)** | | | 3 (<1) | 274 (34) | 130 (38) | 52 (30) | 92 (31) | 0.077 |
| **ECG** | | | | | | | | |
| **Heart Rate (bpm)** | | | 0 (0) | 70 (61 – 80) | 71 (62 – 80) | 69 (61 – 78) | 69 (60 – 80) | 0.23 |
| **Atrial Fibrillation, n. (%)** | | | 0 (0) | 294 (36) | 93 (27) | 56 (32) | 145 (49) | **<0.001** |
| **QRS Width (msec)** | | | 74 (9) | 108 (92 – 138) | 120 (102 – 160) | 110 (94 – 139) | 96 (84 – 114) | **<0.001** |
| **Echocardiography** | | | | | | | | |
| **LVEDD (mm)** | | | 0 (0) | 57 (50 – 63) | 63 (58 – 69) | 58 (52 – 62) | 49 (44 – 53) | **<0.001** |
| **LVEDV (mL)** | | | 0 (0) | 145 (103 – 192) | 191 (156 – 238) | 149 (116 – 178) | 100 (74 – 123) | **<0.001** |
| **LVEF (%)** | | | 0 (0) | 44 (34 – 55) | 32 (26 – 37) | 45 (43 – 46) | 59 (54 – 63) | - |
| **Global Longitudinal Strain (%)** | | | 208 (26) | -10.0 (-13.5 – -6.9) | -6.6 (-8.3 – -4.9) | -10.2 (-11.8 – -8.9) | -14.1 (-17.0 – -12.3) | - |
| **Left Atrial Volume (mL)** | | | 0 (0) | 77 (57 – 104) | 82 (61 – 111) | 70 (55 – 94) | 75 (55 – 101) | **0.004** |
| **LAVI (mL/m^2^)** | | | 0 (0) | 39.7 (29.0 – 54.4) | 44.2 (32.0 – 58.1) | 34.9 (27.1 – 49.3) | 39.2 (28.2 – 54.0) | **0.002** |
| **TAPSE (mm)** | | | 1 (<1) | 18 (15 – 21) | 16 (14 – 20) | 19 (16 – 22) | 19 (16 – 22) | **<0.001** |
| **TR Gradient (mmHg)** | | | 19 (2) | 25 (20 – 33) | 25 (20 – 33) | 23 (18 – 28) | 25 (20 – 34) | **0.017** |
| **IVC Diameter (mm)** | | | 28 (3) | 18 (16 – 22) | 19 (16 – 23) | 17 (16 – 21) | 18 (15 – 23) | **0.015** |
| **Mitral Regurgitation ≥ Mild** | | | 1 (<1) | 385 (47) | 192 (56) | 67 (38) | 126 (43) | **<0.001** |
| **Blood Tests** | | | | | | | | |
| **NTproBNP (ng/L)** | **Overall** | | 1 (<1) | 1116 (423 – 2425) | 1653 (753 – 3667) | 660 (245 – 1759) | 846 (365 – 1882) | **<0.001** |
|  | **SR** | |  | 694 (279 – 1861) | 1345 (600 – 3046) | 364 (182 – 1108) | 402 (239 – 791) | **<0.001** |
|  | **AF** | |  | 1797 (1019 – 3503) | 2326 (1252 – 4131) | 1759 (1063 – 3973) | 1505 (896 – 2520) | **<0.001** |
| **Serum Creatinine (µmol/L)** | | | 0 (0) | 103 (84 – 134) | 106 (87 – 139) | 101 (84 – 127) | 101 (82 – 133) | 0.27 |
| **eGFR (mL/min/1.73 m^2^)** | | | 0 (0) | 57 (40 – 72) | 56 (40 – 72) | 60 (45 – 73) | 55 (39 – 68) | 0.303 |
| **Urea (mmol/L)** | | | 0 (0) | 7.1 (5.4 – 10.0) | 7.3 (5.8 – 10.0) | 6.5 (5.0 – 8.9) | 7.4 (5.4 – 10.7) | **0.012** |
| **Albumin (g/L)** | | | 1 (<1) | 38 (36 – 40) | 38 (36 – 40) | 38 (37 – 40) | 38 (36 – 40) | 0.24 |
| **Haemoglobin (g/dL)** | | **All** | 0 (0) | 13.3 (12.1 – 14.4) | 13.4 (12.3 – 14.3) | 13.5 (12.3 – 14.7) | 13.0 (11.8 – 14.3) | **0.008** |
|  |  | ***Women*** |  | 12.7 (11.6 – 13.7) | 12.7 (11.5 – 13.6) | 12.8 (11.6 – 13.6) | 12.7 (11.5 – 13.8) | 0.89 |
|  |  | ***Men*** |  | 13.6 (12.4 – 14.7) | 13.6 (12.6 – 14.6) | 13.9 (12.6 – 15.2) | 13.4 (12.0 – 14.6) | 0.09 |
| **Iron (µmol/L)** | | | 51 (6) | 14 (11 – 18) | 14 (11 – 18) | 15 (11 – 18) | 14 (10 – 18) | 0.162 |
| **Ferritin (ng/mL)** | | | 42 (5) | 106 (57 – 185) | 106 (56 – 200) | 108 (63 – 195) | 101 (56 – 166) | 0.412 |
| **WBC Count (x10^9^/L)** | | | 0 (0) | 7.0 (5.8 – 8.5) | 7.1 (5.8 – 8.4) | 6.8 (5.8 – 8.5) | 7.1 (5.8 – 8.6) | 0.95 |
| **Neutrophil Count (x10^9^/L)** | | | 0 (0) | 4.38 (3.54 – 5.56) | 4.46 (3.55 – 5.59) | 4.3 (3.5 – 5.4) | 4.4 (3.6 – 5.7) | 0.57 |
| **Lymphocyte Count (x10^9^/L)** | | | 0 (0) | 1.56 (1.19 – 2.06) | 1.51 (1.20 – 1.97) | 1.63 (1.20 – 2.12) | 1.58 (1.16 – 2.11) | 0.38 |
| **Neutrophil-to-Lymphocyte Ratio** | | | 0 (0) | 2.788 (2.004. 3.914) | 2.951 (2.068 – 3.908) | 2.48 (1.92 – 3.91) | 2.79 (2.00 – 4.01) | 0.15 |
| **Monocyte Count (x10^9^/L)** | | | 1 (<1) | 0.630 (0.500 – 0.770) | 0.640 (0.520 – 0.768) | 0.590 (0.480 – 0.780) | 0.650 (0.495 – 0.780) | 0.36 |
| **Eosinophil Count (x10^9^/L)** | | | 0 (0) | 0.170 (0.110 – 0.270) | 0.180 (0.105 – 0.270) | 0.200 (0.120 – 0.310) | 0.150 (0.100 – 0.260) | **0.003** |
| **Basophil Count (x10^9^/L)** | | | 0 (0) | 0.030 (0.020 – 0.040) | 0.030 (0.020 – 0.040) | 0.030 (0.020 – 0.040) | 0.030 (0.020 – 0.040) | 0.65 |
| **hsCRP (mg/L)** | | | 24 (3) | 3.3 (1.5 – 7.1) | 3.0 (1.3 – 6.6) | 3.2 (1.3 – 7.1) | 3.9 (1.8 – 7.7) | 0.05 |
| **Treatment at Time of Referral** | | | | | | | | |
| **Loop Diuretic, n. (%)** | | | 0 (0) | 572 (70) | 261 (76) | 118 (67) | 193 (65) | **0.004** |
| **>40 mg Furosemide/day, n. (%)** | | | 0 (0) | 226 (28) | 106 (31) | 47 (27) | 73 (25) | 0.179 |
| **Beta Blocker, n. (%)** | | | 0 (0) | 619 (76) | 284 (83) | 140 (80) | 195 (66) | **<0.001** |
| **ACEi, n. (%)** | | | 0 (0) | 529 (65) | 249 (73) | 118 (67) | 162 (54) | **<0.001** |
| **ARB, n. (%)** | | | 0 (0) | 161 (20) | 63 (18) | 36 (21) | 62 (21) | 0.719 |
| **MRA, n. (%)** | | | 0 (0) | 274 (34) | 156 (46) | 62 (35) | 56 (19) | **<0.001** |

**Supplementary Table 4.** **Baseline characteristics of patients with heart failure stratified by HF phenotype in a subset of patients with detailed echocardiography**.

Abbreviations used: HF, heart failure; IHD, ischaemic heart disease; COPD, chronic obstructive pulmonary disease; BMI, body mass index; BP, blood pressure; JVP, jugular vein pressure; NYHA, New York Heart Association; HFrEF, heart failure with reduced ejection fraction; HFmrEF, heart failure with mildly reduced ejection fraction; HFpEF, heart failure with preserved ejection fraction; LVEDD, left ventricular end-diastolic diameter; LVEDV, left ventricular end-diastolic volume; LVESV, left ventricular end-systolic volume; LVEF, left ventricular ejection fraction; LAVI, left atrial volume index; TAPSE, tricuspid annular plane systolic excursion; TR gradient, trans-tricuspid systolic gradient; IVC, inferior vena cava; NTproBNP, N-terminal pro–B-type natriuretic peptide; SR, sinus rhythm; AF, atrial fibrillation; eGFR, estimated glomerular filtration rate; WBC, white blood cell; hsCRP, high sensitivity C-reactive protein; ACEi, angiotensin-converting enzyme inhibitor; ARB, angiotensin receptor blocker; MRA, mineralocorticoid receptor antagonist. *Missing refers to missing values from the overall included patients, n = 813.
