# Supplementary material for: Neutrophil‐to‐lymphocyte ratio: link to congestion, inflammation, and mortality in outpatients with heart failure
Source: ESC Heart Fail. 2025 Mar 2;12(3):1571–82. doi: 10.1002/ehf2.15240 (PMC12055385; doi:10.1002/ehf2.15240)
Supplement: Supplementary file 8 — Table S5.Baseline characteristics of patients with heart failure stratified by quartiles of neutrophil‐to‐lymphocyte ratio in a subset of patients with detailed echocardiography. [file EHF2-12-1571-s011.docx]

| **Variable** | | | **Missing*** | **Quartile 1**  **≤ 2.00**  **N= 204** | **Quartile 2**  **2.01 – 2.78**  **N= 203** | **Quartile 3**  **2.79 – 3.91**  **N= 203** | **Quartile 4**  **≥ 3.92**  **N= 203** | **P** |
| --- | --- | --- | --- | --- | --- | --- | --- | --- |
| **Demographics** | | | | | | | | |
| **Age (years)** | | | 0 (0) | 72 (64 – 79) | 73 (65 – 79) | 76 (69 – 81) | 77 (71 – 83) | **<0.001** |
| **Sex (women)** | | | 0 (0) | 72 (35) | 60 (30) | 61 (30) | 60 (30) | 0.53 |
| **Diabetes, n. (%)** | | | 0 (0) | 48 (24) | 61 (30) | 60 (30) | 74 (36) | **0.044** |
| **Hypertension, n. (%)** | | | 0 (0) | 111 (54) | 127 (63) | 107 (53) | 100 (49) | 0.050 |
| **IHD, n. (%)** | | | 0 (0) | 118 (58) | 129 (64) | 125 (62) | 126 (62) | 0.68 |
| **COPD, n. (%)** | | | 0 (0) | 20 (10) | 13 (6) | 28 (14) | 35 (17) | **0.005** |
| **BMI (kg/m^2^)** | | | 0 (0) | 29.7 (26.0 – 33.5) | 29.4 (25.4 – 33.1) | 28.1 (24.1 – 32.3) | 27.1 (24.4 – 30.5) | **<0.001** |
| **Systolic BP (mmHg)** | | | 0 (0) | 130 (114 – 146) | 130 (113 – 146) | 126 (114 – 142) | 121 (106 – 139) | 0.062 |
| **Clinical Examination – Symptoms & Signs** | | | | | | | | |
| **Peripheral Oedema ≥Moderate, n. (%)** | | | 0 (0) | 27 (13) | 34 (17) | 42 (21) | 59 (29) | **<0.001** |
| **Lung Crackles, n. (%)** | | | 0 (0) | 12 (6) | 21 (10) | 30 (15) | 43 (21) | **<0.001** |
| **Raised JVP, n. (%)** | | | 0 (0) | 14 (7) | 15 (7) | 20 (10) | 37 (18) | **<0.001** |
| **Liver Distension, n. (%)** | | | 0 (0) | 8 (4) | 4 (2) | 7 (3) | 20 (10) | **0.001** |
| **NYHA III/IV, n. (%)** | | | 3 (<1) | 49 (24) | 61 (30) | 70 (34) | 94 (46) | **<0.001** |
| **Echocardiography** | | | | | | | | |
| **Heart Rate (bpm)** | | | 0 (0) | 68 (60 – 79) | 69 (60 – 77) | 70 (61 – 79) | 74 (64 – 82) | **<0.001** |
| **Atrial Fibrillation, n. (%)** | | | 0 (0) | 61 (30) | 65 (32) | 79 (39) | 89 (44) | **0.013** |
| **QRS Width (msec)** | | | 74 (9) | 104 (90 – 130) | 108 (93 – 134) | 110 (94 – 148) | 108 (92 – 142) | 0.051 |
| **Blood Tests** | | | | | | | | |
| **HF phenotype** | | **HFrEF** | 0 (0) | 78 (38) | 75 (37) | 106 (52) | 82 (40) | **0.004** |
|  |  | **HFmrEF** |  | 52 (26) | 54 (27) | 25 (12) | 44 (22) |  |
|  |  | **HFpEF** |  | 74 (36) | 74 (36) | 72 (36) | 77 (38) |  |
| **LVEDD (mm)** | | | 0 (0) | 57 (48 – 63) | 57 (49 – 63) | 58 (51 – 65) | 57 (50 – 63) | 0.13 |
| **LVEDV (mL)** | | | 0 (0) | 137 (100 – 187) | 148 (100 – 189) | 153 (111 – 199) | 144 (106 – 193) | 0.063 |
| **LVEF (%)** | | | 0 (0) | 45 (36 – 55) | 44 (35 – 54) | 40 (31 – 54) | 44 (34 – 55) | 0.43 |
| **Global Longitudinal Strain (%)** | | | 208 (26) | -10.6 (-13.4 – -7.5) | -9.7 (-13.6 – -7.1) | -10.0 (-13.6 – -6.8) | -10.1 (-13.5 – -6.0) | 0.48 |
| **Left Atrial Volume (mL)** | | | 0 (0) | 74.5 (56.2 – 98.8) | 68.0 (55.0 – 97.0) | 83.0 (56.0 – 109.0) | 85.0 (60.0 – 114.0) | **<0.001** |
| **LAVI (mL/m^2^)** | | | 0 (0) | 38.4 (28.4 – 50.4) | 35.5 (27.2 – 49.1) | 43.8 (30.7 – 60.2) | 46.3 (32.7 – 60.1) | **<0.001** |
| **TAPSE (mm)** | | | 1 (<1) | 19 (16 – 22) | 19 (16 – 22) | 17 (14 – 21) | 17 (14 – 21) | **<0.001** |
| **TR Gradient (mmHg)** | | | 19 (2) | 22 (18 – 28) | 24 (20 – 31) | 26 (20 – 36) | 28 (20 – 37) | **<0.001** |
| **IVC Diameter (mm)** | | | 28 (3) | 17.5 (16.0 – 21.0) | 17.0 (16.0 – 21.0) | 19.0 (16.0 – 23.0) | 20.0 (16.2 – 24.0) | **<0.001** |
| **Mitral Regurgitation ≥ Mild** | | | 1 (<1) | 92 (45) | 88 (43) | 99 (49) | 106 (52) | 0.28 |
| **Blood Tests** | | | | | | | | |
| **NTproBNP (ng/L)** | **Overall** | | 1 (<1) | 692 (271 – 1641) | 795 (381 – 1818) | 1429 (541 – 2942) | 1886 (753 – 4042) | **<0.001** |
|  | **SR** | |  | 389 (194 – 1194) | 600 (294 – 1156) | 913 (372 – 2224) | 1522 (471 – 2994) | **<0.001** |
|  | **AF** | |  | 1252 (812 – 2064) | 1751 (992 – 2774) | 1878 (1176 – 3518) | 2310 (1070 – 4558) | **0.001** |
| **Serum Creatinine (µmol/L)** | | | 0 (0) | 92 (81 – 112) | 108 (84 – 141) | 103 (84 – 126) | 120 (90 – 159) | **<0.001** |
| **eGFR (mL/min/1.73 m^2^)** | | | 0 (0) | 63 (52 – 75) | 56 (39 – 73) | 58 (43 – 71) | 46 (34 – 66) | **<0.001** |
| **Urea (mmol/L)** | | | 0 (0) | 6.4 (5.0 – 7.7) | 7.3 (5.2 – 10.1) | 7.3 (5.5 – 9.8) | 8.8 (6.2 – 12.3) | **<0.001** |
| **Albumin (g/L)** | | | 1 (<1) | 39 (37 – 41) | 39 (37 – 41) | 38 (36 – 40) | 37 (35 – 39) | **<0.001** |
| **Haemoglobin (g/dL)** | | **All** | 0 (0) | 13.6 (12.6 – 14.6) | 13.6 (12.4 – 14.8) | 13.4 (12.2 – 14.3) | 12.6 (11.3 – 13.8) | **<0.001** |
|  |  | ***Women*** |  | 13.0 (11.8. 13.6) | 12.8 (11.8 – 14.0) | 12.4 (11.5 – 13.7) | 12.0 (10.6 – 13.6) | **0.012** |
|  |  | ***Men*** |  | 14.1 (13.1 – 15.0) | 13.9 (12.7 – 15.1) | 13.6 (12.6 – 14.5) | 12.8 (11.6 – 13.8) | **<0.001** |
| **Iron (µmol/L)** | | | 51 (6) | 16 (13 – 20) | 15 (12 – 18) | 14 (11 – 18) | 11 (9 – 15) | **<0.001** |
| **Ferritin (ng/mL)** | | | 42 (5) | 90 (53 – 189) | 105 (58 – 197) | 110 (64 – 201) | 112 (60 – 169) | 0.703 |
| **WBC Count (x10^9^/L)** | | | 0 (0) | 6.4 (5.3 – 7.6) | 7.2 (6.1 – 8.5) | 6.9 (5.9 – 8.5) | 7.7 (6.3 – 9.3) | **<0.001** |
| **Neutrophil Count (x10^9^/L)** | | | 0 (0) | 3.4 (2.7 – 4.1) | 4.4 (3.6 – 5.1) | 4.6 (3.8 – 5.8) | 5.7 (4.5 – 7.0) | - |
| **Lymphocyte Count (x10^9^/L)** | | | 0 (0) | 2.13 (1.78 – 2.66) | 1.89 (1.50 – 2.24) | 1.40 (1.19 – 1.69) | 1.01 (0.81 – 1.29) | - |
| **Monocyte Count (x10^9^/L)** | | | 1 (<1) | 0.590 (0.460 – 0.725) | 0.630 (0.510 – 0.810) | 0.640 (0.510 – 0.760) | 0.670 (0.530 – 0.810) | **0.002** |
| **Eosinophil Count (x10^9^/L)** | | | 0 (0) | 0.180 (0.110 – 0.290) | 0.170 (0.110 – 0.280) | 0.190 (0.110 – 0.270) | 0.150 (0.090 – 0.260) | **0.041** |
| **Basophil Count (x10^9^/L)** | | | 0 (0) | 0.030 (0.020 – 0.040) | 0.030 (0.020 – 0.040) | 0.030 (0.020 – 0.040) | 0.030 (0.020 – 0.040) | 0.15 |
| **hsCRP (mg/L)** | | | 24 (3) | 2.2 (1.0 – 4.8) | 2.6 (1.4 – 5.5) | 3.2 (1.5 – 7.6) | 6.4 (3.1 – 13.8) | **<0.001** |
| **Treatment at Time of Referral** | | | | | | | | |
| **Loop Diuretic, n. (%)** | | | 0 (0) | 123 (60) | 135 (66) | 155 (76) | 159 (78) | **<0.001** |
| **>40 mg Furosemide/day, n. (%)** | | | 0 (0) | 37 (18) | 51 (25) | 56 (28) | 82 (40) | **<0.001** |
| **Beta Blocker, n. (%)** | | | 0 (0) | 161 (79) | 163 (80) | 149 (73) | 146 (72) | 0.13 |
| **ACEi, n. (%)** | | | 0 (0) | 132 (65) | 136 (67) | 126 (62) | 135 (66) | 0.72 |
| **ARB, n. (%)** | | | 0 (0) | 42 (21) | 44 (22) | 39 (19) | 36 (18) | 0.77 |
| **MRA, n. (%)** | | | 0 (0) | 71 (35) | 68 (34) | 68 (34) | 67 (33) | 0.98 |

**Supplementary Table 5**. **Baseline characteristics of patients with heart failure stratified by quartiles of neutrophil-to-lymphocyte ratio in a subset of patients with detailed echocardiography.** Abbreviations used: HF, heart failure; IHD, ischaemic heart disease; COPD, chronic obstructive pulmonary disease; BMI, body mass index; BP, blood pressure; JVP, jugular vein pressure; NYHA, New York Heart Association; HFrEF, heart failure with reduced ejection fraction; HFmrEF, heart failure with mildly reduced ejection fraction; HFpEF, heart failure with preserved ejection fraction; LVEDD, left ventricular end-diastolic diameter; LVEDV, left ventricular end-diastolic volume; LVESV, left ventricular end-systolic volume; LVEF, left ventricular ejection fraction; LAVI, left atrial volume index; TAPSE, tricuspid annular plane systolic excursion; TR gradient, trans-tricuspid systolic gradient; IVC, inferior vena cava; NTproBNP, N-terminal pro–B-type natriuretic peptide; SR, sinus rhythm; AF, atrial fibrillation; eGFR, estimated glomerular filtration rate; WBC, white blood cell; hsCRP, high sensitivity C-reactive protein; ACEi, angiotensin-converting enzyme inhibitor; ARB, angiotensin receptor blocker; MRA, mineralocorticoid receptor antagonist. *Missing refers to missing values from the overall included patients, n = 813.
