# Supplementary material for: Neutrophil‐to‐lymphocyte ratio: link to congestion, inflammation, and mortality in outpatients with heart failure
Source: ESC Heart Fail. 2025 Mar 2;12(3):1571–82. doi: 10.1002/ehf2.15240 (PMC12055385; doi:10.1002/ehf2.15240)
Supplement: Supplementary file 11 — Table S8. Rate, cause, and mode of death at 2 years by quartile of NLR according to different HF phenotypes. [file EHF2-12-1571-s003.docx]

| **Q1 ≤ 2.05** | **HFrEF** | | | | | **HFmrEF** | | | | | **HFpEF** | | | | |
| --- | --- | --- | --- | --- | --- | --- | --- | --- | --- | --- | --- | --- | --- | --- | --- |
| **Q2 2.06 – 2.89** |  |  |  |  |  |  |  |  |  |  |  |  |  |  |  |
| **Q3 2.90 – 4.09** | **All**  **1604** | **Q1**  **366** | **Q2**  **367** | **Q3**  **442** | **Q4**  **429** | **All**  **966** | **Q1**  **237** | **Q2**  **260** | **Q3**  **224** | **Q4**  **245** | **All**  **2115** | **Q1**  **569** | **Q2**  **545** | **Q3**  **506** | **Q4**  **495** |
| **Q4 ≥ 4.10** |  |  |  |  |  |  |  |  |  |  |  |  |  |  |  |
| **2-year Mortality** | **366 (23)** | **43 (12)** | **65 (18)** | **97 (22)** | **161 (38)** | **198 (20)** | **26 (11)** | **42 (16)** | **53 (24)** | **77 (31)** | **387 (18)** | **50 (9)** | **80 (15)** | **98 (19)** | **159 (32)** |
| **Cardiovascular** | **241 (66)** | **27 (63)** | **43 (66)** | **60 (62)** | **111 (69)** | **106 (54)** | **16 (62)** | **26 (62)** | **31 (58)** | **33 (43)** | **177 (46)** | **20 (40)** | **46 (57)** | **44 (45)** | **67 (42)** |
| Sudden | 116 (32) | 11 (25) | 23 (35) | 33 (34) | 49 (31) | 63 (32) | 8 (31) | 17 (41) | 20 (38) | 18 (23) | 95 (25) | 12 (24) | 23 (28) | 24 (25) | 36 (22) |
| Terminal HF | 92 (25) | 8 (19) | 17 (26) | 20 (21) | 47 (29) | 25 (13) | 3 (12) | 6 (14) | 7 (13) | 9 (12) | 52 (13) | 7 (14) | 12 (15) | 11 (11) | 22 (14) |
| Other/CV | 33 (9) | 8 (19) | 3 (5) | 7 (7) | 15 (9) | 18 (9) | 5 (19) | 3 (7) | 4 (7) | 6 (8) | 30 (8) | 1 (2) | 11 (14) | 9 (9) | 9 (6) |
| **Non-CV** | **120 (33)** | **16 (37)** | **21 (32)** | **34 (35)** | **49 (30)** | **86 (43)** | **10 (38)** | **15 (36)** | **20 (38)** | **41 (53)** | **202 (52)** | **30 (60)** | **30 (38)** | **54 (55)** | **88 (55)** |
| Infection | 50 (14) | 7 (16) | 10 (15) | 17 (18) | 16 (10) | 29 (15) | 3 (11) | 7 (17) | 3 (6) | 16 (21) | 92 (24) | 16 (32) | 8 (10) | 22 (23) | 46 (29) |
| Cancer | 38 (10) | 5 (12) | 6 (9) | 9 (9) | 18 (11) | 32 (16) | 5 (19) | 7 (17) | 8 (15) | 12 (15) | 47 (12) | 4 (8) | 11 (14) | 13 (13) | 19 (12) |
| Other/Non-CV | 32 (9) | 4 (9) | 5 (8) | 8 (8) | 15 (9) | 25 (12) | 2 (8) | 1 (2) | 9 (17) | 13 (17) | 63 (16) | 10 (20) | 11 (14) | 19 (19) | 23 (14) |
| **Unknown** | **5 (1)** | **0 (0)** | **1 (2)** | **3 (3)** | **1 (1)** | **6 (3)** | **0 (0)** | **1 (2)** | **2 (4)** | **3 (4)** | **8 (2)** | **0 (0)** | **4 (5)** | **0 (0)** | **4 (3)** |
| **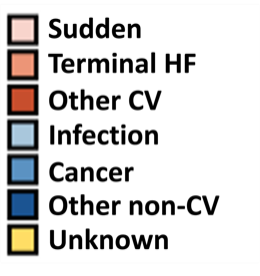** | **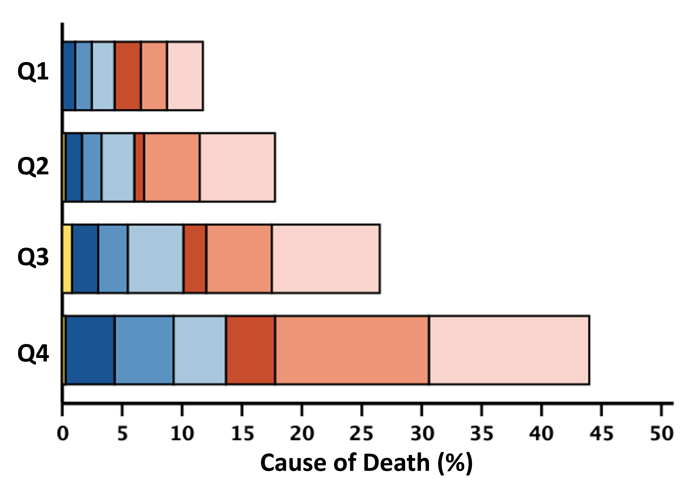.**  **.**  **.**  **.**  **.**  **.**  **.**  **.** | | | | | **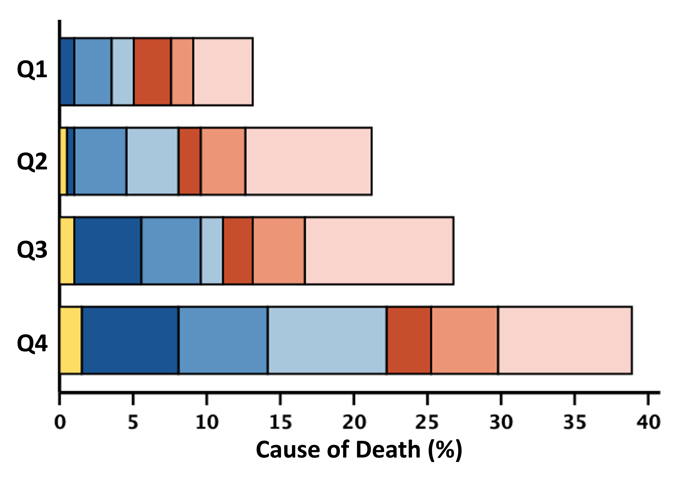** | | | | | **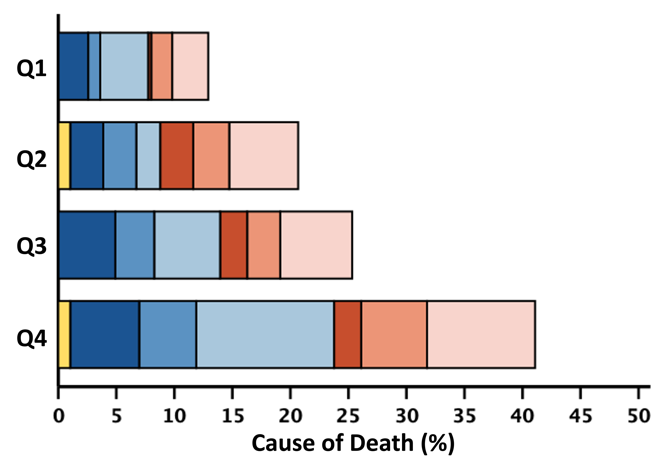** | | | | |

**Supplementary Table 8. Rate, cause, and mode of death at 2 years by quartile of NLR according to different HF phenotypes.**

2-year all-cause mortality expressed as a percentage of the total number of patients overall, and in respective quartiles. Cause of death rows represent the percentage of the total number of deaths in patients overall and in respective quartiles. The stacked bar figures show cause /mode of death by quartile of white cell variable, represented as a percentage of the total number of deaths in the overall population. Abbreviations used: HF, heart failure; CV, cardiovascular.
